# One Health monitoring reveals invasive freshwater snail species, new records, and undescribed parasite diversity in Zimbabwe

**DOI:** 10.1186/s13071-024-06307-4

**Published:** 2024-05-22

**Authors:** Aspire Mudavanhu, Ruben Schols, Emilie Goossens, Tamuka Nhiwatiwa, Tawanda Manyangadze, Luc Brendonck, Tine Huyse

**Affiliations:** 1 Department of Biological Sciences, Bindura University of Science Education, Bindura, Zimbabwe; 2 Laboratory of Animal Ecology, Global Change and Sustainable Development, KU Leuven, Leuven, Belgium; 3 Department of Biology, Royal Museum for Central Africa, Tervuren, Belgium; 4 Laboratory of Aquatic Biology, KU Leuven Kulak, Kortrijk, Belgium; 5 Department of Fisheries and Ocean Sciences, School of Agriculture and Fisheries, University of Namibia, Henties Bay, Namibia; 6 Department of Geosciences, School of Geosciences, Disaster and Development, Faculty of Science and Engineering, Bindura University of Science Education, Bindura, Zimbabwe; 7 Discipline of Public Health Medicine, College of Health Sciences, University of KwaZulu-Natal, Durban, 4000 South Africa; 8 Water Research Group, Unit for Environmental Sciences and Management, North-West University, Potchefstroom, South Africa

**Keywords:** Gastropoda, Trematodiases, Xenomonitoring, One Health, Schistosomiasis, Invasive species, Barcoding void, Exotic snail species

## Abstract

**Background:**

Snail-borne trematodes afflict humans, livestock, and wildlife. Recognizing their zoonotic potential and possible hybridization, a One Health approach is essential for effective control. Given the dearth of knowledge on African trematodes, this study aimed to map snail and trematode diversity, focusing on (i) characterizing gastropod snail species and their trematode parasites, (ii) determining infection rates of snail species as intermediate hosts for medically, veterinary, and ecologically significant trematodes, and (iii) comparing their diversity across endemic regions.

**Methods:**

A cross-sectional study conducted in 2021 in Chiredzi and Wedza districts in Zimbabwe, known for high human schistosomiasis prevalence, involved malacological surveys at 56 sites. Trematode infections in snails were detected through shedding experiments and multiplex rapid diagnostic polymerase chain reactions (RD-PCRs). Morphological and molecular analyses were employed to identify snail and trematode species.

**Results:**

Among 3209 collected snail specimens, 11 species were identified, including schistosome and fasciolid competent snail species. We report for the first time the invasive exotic snail *Tarebia granifera* in Zimbabwe, which was highly abundant, mainly in Chiredzi, occurring at 29 out of 35 sites. Shedding experiments on 1303 snails revealed a 2.24% infection rate, with 15 trematode species identified through molecular genotyping. Five species were exclusive to Chiredzi: *Bolbophorus* sp., *Schistosoma mansoni*, *Schistosoma mattheei, Calicophoron* sp., and *Uvulifer* sp. Eight were exclusive to Wedza, including *Trichobilharzia* sp., *Stephanoprora amurensis*, *Spirorchid* sp., and *Echinostoma* sp. as well as an unidentified species of the Plagiorchioidea superfamily. One species, *Tylodelphys mashonensis*, was common to both regions. The RD-PCR screening of 976 non-shedding snails indicated a 35.7% trematode infection rate, including the presence of schistosomes (1.1%) *Fasciola nyanzae* (0.6%). In Chiredzi, *Radix natalensis* had the highest trematode infection prevalence (33.3%), while in Wedza, *R. natalensis* (55.4%) and *Bulinus tropicus* (53.2%) had the highest infection prevalence.

**Conclusions:**

Our xenomonitoring approach unveiled 15 trematode species, including nine new records in Zimbabwe. *Schistosoma mansoni* persists in the study region despite six mass deworming rounds. The high snail and parasite diversity, including the presence of exotic snail species that can impact endemic species and biomedically important trematodes, underscores the need for increased monitoring.

**Graphical Abstract:**

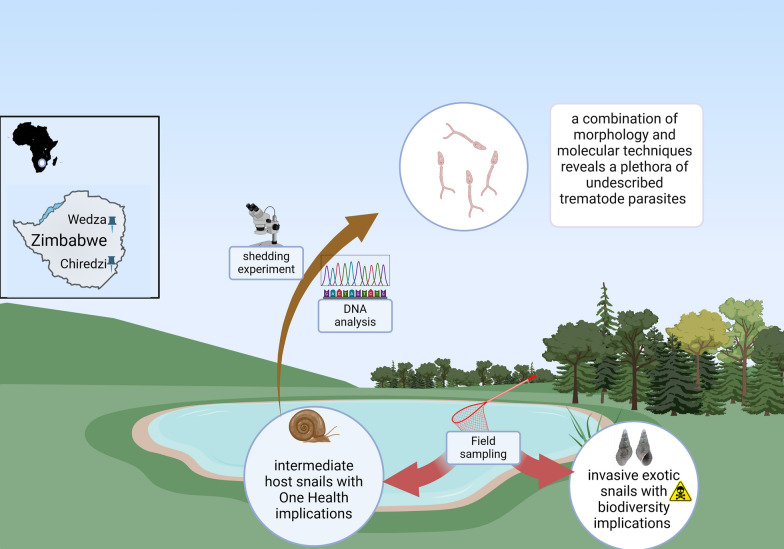

**Supplementary Information:**

The online version contains supplementary material available at 10.1186/s13071-024-06307-4.

## Background

Millions of people in approximately 90 countries suffer from snail-borne trematodiases [1, 2]. For example, schistosomiasis, a widespread and neglected tropical disease caused by species of the genus *Schistosoma*, holds significant prevalence, morbidity, and socioeconomic implications [2]. Moreover, trematodiases like schistosomiasis, fasciolosis, echinostomiasis, and amphistomosis also form an economic burden on the livestock industry as they lead to production losses and mortality in livestock [3]. For example, fasciolosis alone causes global economic losses of over US$3.2 billion per year [4]. Although the impact of trematodes on wildlife populations is poorly understood, high parasite load may lead to mortality which threatens conservation efforts [5, 6].

Some trematodiases are regarded as zoonotic. Fasciolosis is primarily of concern in domestic and wild animals, but human cases have been reported in 42 countries, mostly in Asia and South America [7–9]. *Fasciola* hybrids commonly occur and are capable of infecting humans [10, 11]. Hybridization between human and animal schistosome species has also been reported [12–14], demonstrating the importance of animal reservoirs in disease ecology. Therefore, it is recommended to study trematodiases within a One Health framework, where the health of humans, livestock, and wildlife is considered intrinsically linked and equally important for mapping, detecting high-risk locations, and improving disease control [13, 15–17].

In Zimbabwe, trematodiases due to schistosomes, fasciolids, and amphistomes have been reported [18–22]. The presence of human schistosomiasis in Zimbabwe has long been documented [23–25] and the latest national survey revealed the disease is present in at least 91% of all districts, being most prevalent in Chiredzi, Wedza, and Shamva districts [24]. Human fasciolosis has also been described in Zimbabwe [26], but no recent cases have been reported. Records of animal fasciolosis due to *Fasciola gigantica* in livestock [27], *Fasciola hepatica* in antelope (*Hippotragus niger*) and a duiker (*Sylvicapra grimmia*) [19] and recently *Fasciola nyanzae* in the common hippopotamus [28] have been published.

Recent malacological surveys in the country revealed a high diversity of trematodes [28–31], many of which could not be identified through molecular barcoding techniques because of the gross underrepresentation of trematode reference sequences in public databases, the so-called “barcoding void” [30]. Failure to identify trematodes hinders prompt interventions, further reinforcing their neglected status.

For this study, the Chiredzi and Wedza districts in Zimbabwe were chosen because of reported high schistosomiasis prevalence in 2014 [24]. Revisited after a decade, we extended our study to all trematode species and their associated freshwater snail species, aligning with the One Health approach. This study sought to (i) characterize gastropod snail and trematode diversity and (ii) determine infection rates in snails acting as intermediate hosts for medically, veterinary, and ecologically significant trematodes.

## Methods

### Study area

The cross-sectional malacological survey was conducted in August 2021, during which 35 sites in Chiredzi and 21 in Wedza were sampled. Chiredzi, in southeastern Zimbabwe, is renowned for sugarcane farming, notably around the Chiredzi and Runde rivers, with major plantations at Hippo Valley Estates, Triangle, and Mkwasine. The region's irrigation relies on canal-facilitated flooding. With mean annual rainfall of 582 mm, Chiredzi is semi-arid, experiencing a hot-wet season (mid-November to April), cool-dry season (May to late July), and hot-dry season (August to mid-November). Daily temperatures frequently surpass 32 °C, reaching peaks of at least 45 °C, making it a water-stressed region [32]. Over 70% of Chiredzi is dedicated to wildlife conservation, with Gonarezhou National Park (GNP), Malilangwe Wildlife Reserve (MWR), and Save Conservancy as principal custodians.

Wedza, on the other hand, has a temperate highland tropical climate that typically receives about 675 mm of precipitation spread across 143 rainy days annually [32]. The annual average temperature is 21.5 °C. The district is bound by the Save River in the west and the Ruzave River in the east. Other rivers include Chineyi, Mhare, Nyamidzi, and Nyamhembe.

In Chiredzi, we focused on the protected area of MWR and human-impacted regions, including Chipimbi, Hippo Valley, and Triangle. In Wedza district, the primary focus was on the Imire Rhino and Wildlife Conservation area (referred to as Imire), dedicated to wildlife preservation and partially to tobacco farming. Selection of sampling sites in both regions was based on features indicative of potential transmission areas, including areas with frequent human and/or animal water contact. Refer to Additional file 1: Table S1 for detailed site characteristics.

### Field collection of snails and trematodes

Sampling occurred in a standardized way across all sites, by scooping for 30 min by two persons per site with a scoop net of 1-mm mesh size with a 2-m-long handle. GPS coordinates were recorded at each sampling point. Live snails were transported in ambient water to the field laboratory for sorting, counting, and identification to species or genus level using keys by Mandahl-Barth [33], Brown [34], and Appleton & Miranda [35]. Snails were put in 24-well tissue culture plates with rested borehole water and kept in the dark overnight as per Gumble et al. [36]. The morning after, the snails were exposed to light for several hours and visually checked for cercariae. Emerging cercariae were individually isolated on microscopic slides and photographed and measured using BMS camera (model number: XFCAM1080PHD) and associated BMS pix3 software (version 3.7.8942.20170412) with attention on morphological traits (i.e. oral and ventral suckers, stylet, eyespots, collar spines, tail, and finfold) to classify them per morphotype using keys by Frandsen & Christensen [37]. Shedding snails and cercariae were individually stored, while non-shedding snails were pooled by species per site in 80% ethanol in larger 50-ml ethanol tubes for transportation to Belgium for molecular analysis.

### Snail and trematode DNA extraction

A sterile needle was used to make an incision in the shell apex and subsequently push the soft tissue out of the shell. Next, the isolated tissue was dried using absorbent paper. DNA was extracted using the E.Z.N.A.^®^ Mollusc DNA Kit (OMEGA Bio-Tek, Norcross, GA, USA) according to the manufacturer’s protocol and eluted through two elution steps of 50 μl, totaling 100 μl of DNA extract. Only medical and veterinary important snails including planorbids and lymnaeids were subjected to DNA extraction as they would later be examined for infection using PCR (see next section). For each of the remaining snail species, one specimen per species was extracted for phylogenetic analyses.

The DNA of individual cercariae was extracted using a proteinase *K*-based lysis buffer as described in Ziętara et al. [38]. In short, 10 μl of the prepared proteinase *K* lysis buffer was added to a single cercaria in 10 μl of MilliQ water (Merck, Darmstadt, Germany). Next, a two-step incubation followed at 65 °C for 25 min and at 95 °C for 10 min after which DNA extracts were stored undiluted at − 20 °C.

### Molecular infection diagnosis in snails

Up to 30 specimens of biomedically important snails (i.e. belonging to the *Bulinus* and *Biomphalaria* genera or the family of the Lymnaeidae) were subsampled per species, per site for DNA extraction and tested for infection through the multiplex rapid diagnostic polymerase chain reaction (RD-PCR) methods described by Schols et al. [39] and Carolus et al. [29]. These approaches enable the detection of (pre)patent infections of any trematode species, including *Schistosoma* and *Fasciola* species, in snail DNA extracts. Briefly, both methods are based on the amplification of multiple markers of different lengths in a single PCR reaction: an internal control that amplifies snail 18S rDNA to confirm DNA extraction and PCR success, a general trematode primer pair designed to amplify an 18S rDNA fragment of all trematode genera that have a reference sequence available in GenBank, and primers for *Schistosoma* or *Fasciola*- specific amplification based on the internal transcribed spacer 2 (ITS2) and nuclear-repeat region respectively. All used primers and PCR protocols are described elsewhere [29, 39]. Samples that were positive for *Schistosoma* spp. infection were subsequently analyzed using a species-specific multiplex PCR according to the two-step approach described by Schols et al. [39]. This multiplex PCR differentiates between several schistosomes of veterinary and medical importance (i.e. *Schistosoma haematobium*, *S. mansoni*, *S. mattheei*, and *S. bovis*/*S. curassoni*/*S. guineensis*) by generating species-specific COI amplicons of different lengths.

### PCR and DNA sequencing of snails and trematodes

Snails were identified using the COI marker whereas trematodes were identified by targeting the COI, 18S rDNA, and the ribosomal internal transcribed spacer (ITS) 1 and 2 markers. The primers and resulting amplicon lengths are listed in Table 1, together with those for snail and trematode identification. PCR assays were run in a 20 μl reaction volume using the Qiagen™ Taq DNA polymerase kit comprising 0.6 mM dNTP mix, 2 µl 10 × PCR buffer, 1.5 mM MgCl_2_, 0.12 µl Taq Polymerase at 5 units/µl, 0.8 μM of each primer, and 2 μl DNA extract (1:10 diluted). The PCR cycling parameters varied depending on the respective primers used following Schols et al. [28], and the PCR amplifications were performed in a Biometra^®^ Tprofessional Thermal Cycler. PCR products were visualized by gel electrophoresis on a 2% agarose gel containing Midori Green Direct^®^ staining using a UV transilluminator. Samples exhibiting a clear and bright PCR product were purified using the ExoSAP (Fermentas™) PCR purification protocol and sequenced using the BigDye^®^ chemistry by Macrogen™.

**Table 1:** Primers used to obtain mitochondrial (COI) and nuclear (18S, ITS1, 5.8S, ITS2, and 28S) amplicons for sequencing

Target organism	Primer name	Marker	Length (bp)	Oligonucleotide sequence (5′–3′)	References
Snails	COI_snail_F	COI	563	TAATGTWATTGTTACAGCACATGC	Hammoud et al. [96]
Snails	COI_snail_R	COI	563	GTTGRTATAAAATAGGATCACCWCC	Hammoud et al. [96]
Cercariae	COI1_dig_F	COI	943	CNATGATNTTNTTTTTTTTRATGCC	Hammoud et al. [96]
Cercariae	Nasmit R	COI	943	ACATAATGAAARTCAGCNAYMACRA	Hammoud et al. [96]
Snail infections	COI1_dig_F	COI	451	CNATGATNTTNTTTTTTTTRATGCC	Hammoud et al. [96]
Snail infections	COI1_dig_R	COI	451	GMASWACCAAAWTTHCGATCAAA	Schols et al. [28]
Cercaria and snail infections	WormA	18S rDNA	1870	GCGAATGGCTCATTAAATCAG	Waeschenbach et al. [97]
Cercaria and snail infections	WormB	18S rDNA	1870	CTTGTTACGACTTTTACTTCC	Waeschenbach et al. [97]
Cercaria and snail infections	1270R	18S rDNA	1870	CCGTCAATTCCTTTAAGT	Littlewood & Olson [98]
Cercaria and snail infections	ITS5	ITS1-5.8S-ITS2	1065	GGAAGTAAAAGTCGTAACAAG	White et al. [99]
Cercaria and snail infections	ITS4	ITS1-5.8S-ITS2	1065	TCCTCCGCTTATTGATATGC	White et al. [99]

The target organism is listed (i.e. “snails,” released “cercaria,” and “snail infections” uncovered with RD-PCR) with the primer name, the targeted marker, the annealing temperature, the amplicon length, the primer sequence, and the literature reference from which primers were obtained

### Phylogenetic analysis

Sequences were processed in Geneious Prime^®^ (version 2023.1.1), carefully addressing ambiguities by examining complete chromatograms before and after consensus generation to ensure optimal sequence quality. The Basic Local Alignment Search Tool (BLAST) of the National Center for Biotechnology Information (NCBI) (https://blast.ncbi.nlm.nih.gov/) was used to probe species identity for all sequences of sufficient quality. Sequences with ambiguous species identity (pairwise distance > 5%, and various species in top BLAST results) were further analyzed through phylogenetic reconstructions with sequences from closely related taxa. Those sequences were mined from GenBank and aligned with MAFFT v.7 implemented online (https://mafft.cbrc.jp/alignment/server; [40]) with default settings. As an extra step, the least stringent settings in Gblocks available online at NGPhylogeny.fr (https://ngphylogeny.fr/; [41]) were applied to all ITS and 18S rDNA alignments. The model selection analysis was run in MEGA (version 11.0.13) to evaluate the most suitable substitution model based on the Akaike information criterion (AIC) and Bayesian information criterion (BIC) values from the model list. The suitable substitution models for all alignments and phylogenetic analyses performed in this study are described in the captions of their respective tree figures and supplementary files.

Lastly, the IQ-tree web servers (http://iqtree.cibiv.univie.ac.at) were used to calculate a maximum likelihood (ML) tree, and nodal support was assessed by performing 10,000 bootstrap replicates [42]. The resulting trees were rooted with the respective outgroups in Figtree (version 1.4.4) and exported to TreeGraph 2 (version 2.15.0–887 beta) [43] where nodes with support values < 70% were collapsed. The final tree was edited in Inkscape (version 1.3) where ill-placed nodal support values were adjusted, and species names were italicized.

## Results

### Observed freshwater snail species

**Fig. 1:**
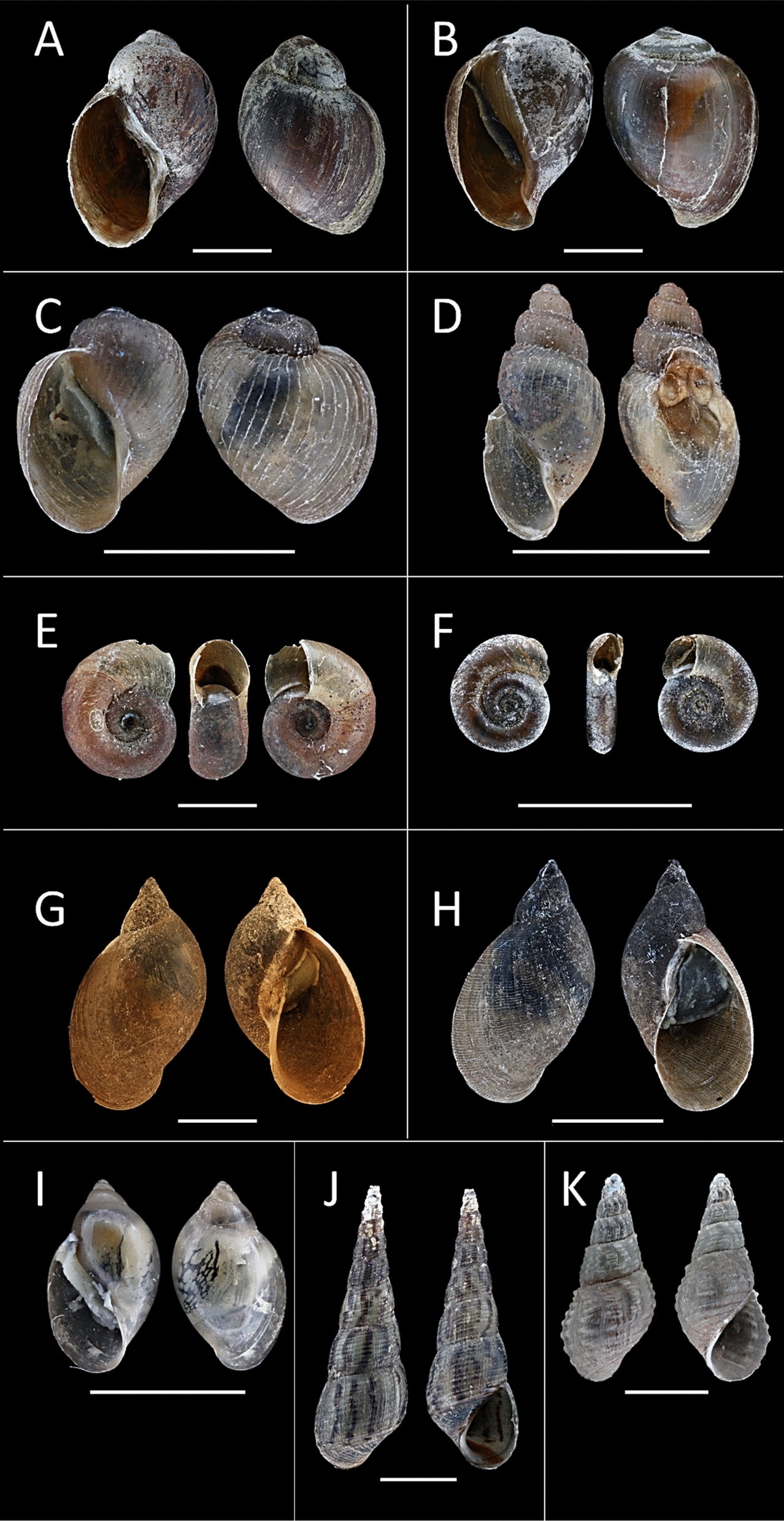
All 11 snail species sampled in Chiredzi and Wedza districts of Zimbabwe. Photographs were captured using a Canon EOS 600D camera with a Macro Photo Lens to capture images of specimens that were fixed on a dark clay platform presented in apertural, lateral, and sometimes apical view. The images were stacked using Zerene Stacker™ software and processed in Adobe Photoshop^®^, removing the background and combining the front and rear perspectives into one picture with a consistent scale size. **A** *Bulinus tropicus*; **B** *B. globosus*; **C** *B. truncatus*; **D** *B. forskalii*; **E** *Biomphalaria pfeifferi*; **F** *Gyraulus* sp.; **G** *Radix natalensis*; **H** *Pseudosuccinea columella*; **I** *Physella acuta*; **J** *Melanoides tuberculata*; **K** *Tarebia granifera*. The white line beneath the photo of each snail represents a scale bar of 5 mm

**Fig. 2:**
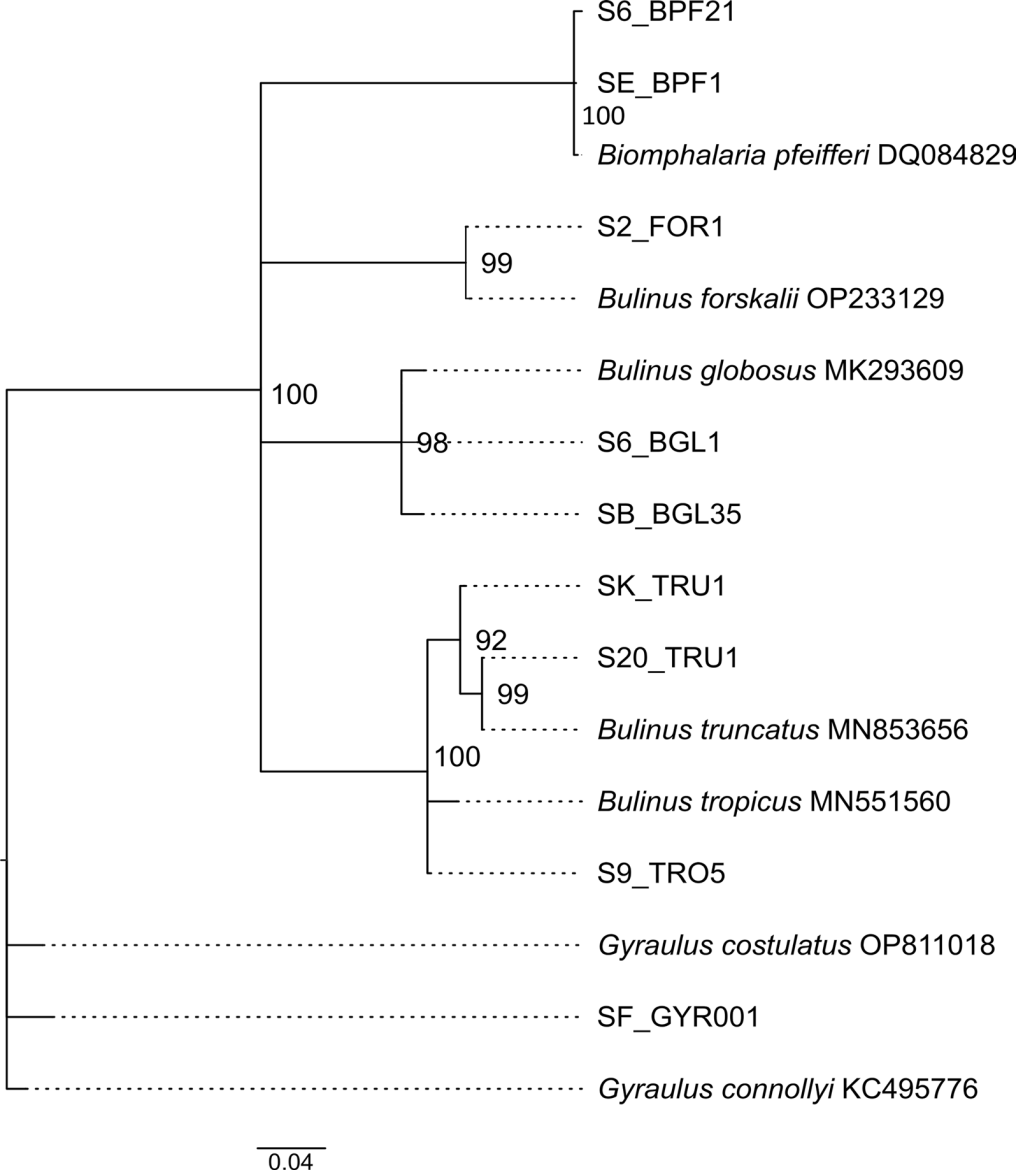
Maximum likelihood phylogenetic trees of the family Planorbidae using COI (655 bp) sequences and using the General Time Reversible (GTR) model [95] with discrete gamma distribution ([+ G] = 0.24). Bootstrap values (10,000 replicates) > 70 are shown next to the branches. GenBank sequences are displayed with their accession number (not italicized). Sequences without accession number were obtained during this study and can be linked to pictures of freshwater snails shown in the Fig. 1. The number or letter after the S-prefix denotes site name and respective the code name is given (BPF = *B. pfeifferi*, FOR = *B. forskalii*, BGL = *B. globosus*, TRU = *B. truncatus*, TRO = *B. tropicus*, and GYR = *Gyraulus* sp.)

Eleven different species of freshwater snails were observed from both Chiredzi and Wedza: *Gyraulus* sp., *Melanoides tuberculata*, *Tarebia granifera*, *Biomphalaria pfeifferi*, *Pseudosuccinea columella*, *Physella acuta*, *Radix natalensis*, *Bulinus globosus*, *B. truncatus*, *B. forskalii*, and *B. tropicus* (Fig. 1). The COI-based BLAST results from all our freshwater snails were satisfactorily robust for species identification: BLAST identity ≥ 99% with query cover of 100%, sequence lengths of + 300 bp, and HQ ≥ 95%. Our 467-bp *B. pfeifferi* sequences from both Chiredzi and Wedza had 100% nucleotide similarity with Malawian specimens (“GenBank: OR880274”). Similarly, a 100% match was observed between our 483 bp *T. granifera* sequences and those reported from China (“GenBank: MZ662113”) and Thailand (“GenBank: MK000375”). The sequences of our *R. natalensis* (467 bp) from Wedza matched perfectly with previous Zimbabwean records at least 150 km up north in Mazowe Dam (Mazowe district) and Mwenje Dam (Chiweshe district) (“GenBank: MT992958”). Our *P. columella* sequences (483 bp) indicated 100% similarity with records of the invasive haplotype across Africa (South Africa: “GenBank: MN601428” and Egypt: “GenBank: LC015522”), Australia (“GenBank: AY227366”), Europe (Spain: “GenBank: KP242798”), and North America (New Mexico, USA: “GenBank: NC_042905”). Similarly, our *P. acuta* sequence of 314 bp was identical to submissions from Asia (Japan: “GenBank: LC429395”), the Middle East (Iran: “GenBank: KT280442”), and the Americas (New York, USA: “GenBank: MN164018”). Our *M. tuberculata* sequence was of low quality (HQ 88.6%); hence, only morphology was used. The Planorbidae reference sequences from GenBank overlapped at least 378 bp with our partially sequenced COI marker. Using this fragment in phylogenetic inference, the species status and evolutionary relationships of *B. globosus*, *B. truncatus*, *B. forskalii*, and *B. tropicus* specimens collected in Chiredzi and Wedza were confirmed (Fig. 2). The smallest COI pairwise distances between our *Gyraulus* sp. (439 bp) and the reference sequences from GenBank were 3.2% (*G. costulatus* from Senegal “GenBank: OP811018”) and 4% (*G. connollyi* from South Africa “GenBank: KC495776”) (results not shown). However, our specimen clustered with *G. costulatus* (from Senegal “GenBank: OP811018”) in the phylogenetic tree (Fig. 2).

### Abundance and spatial distribution of freshwater snails

**Fig. 3:**
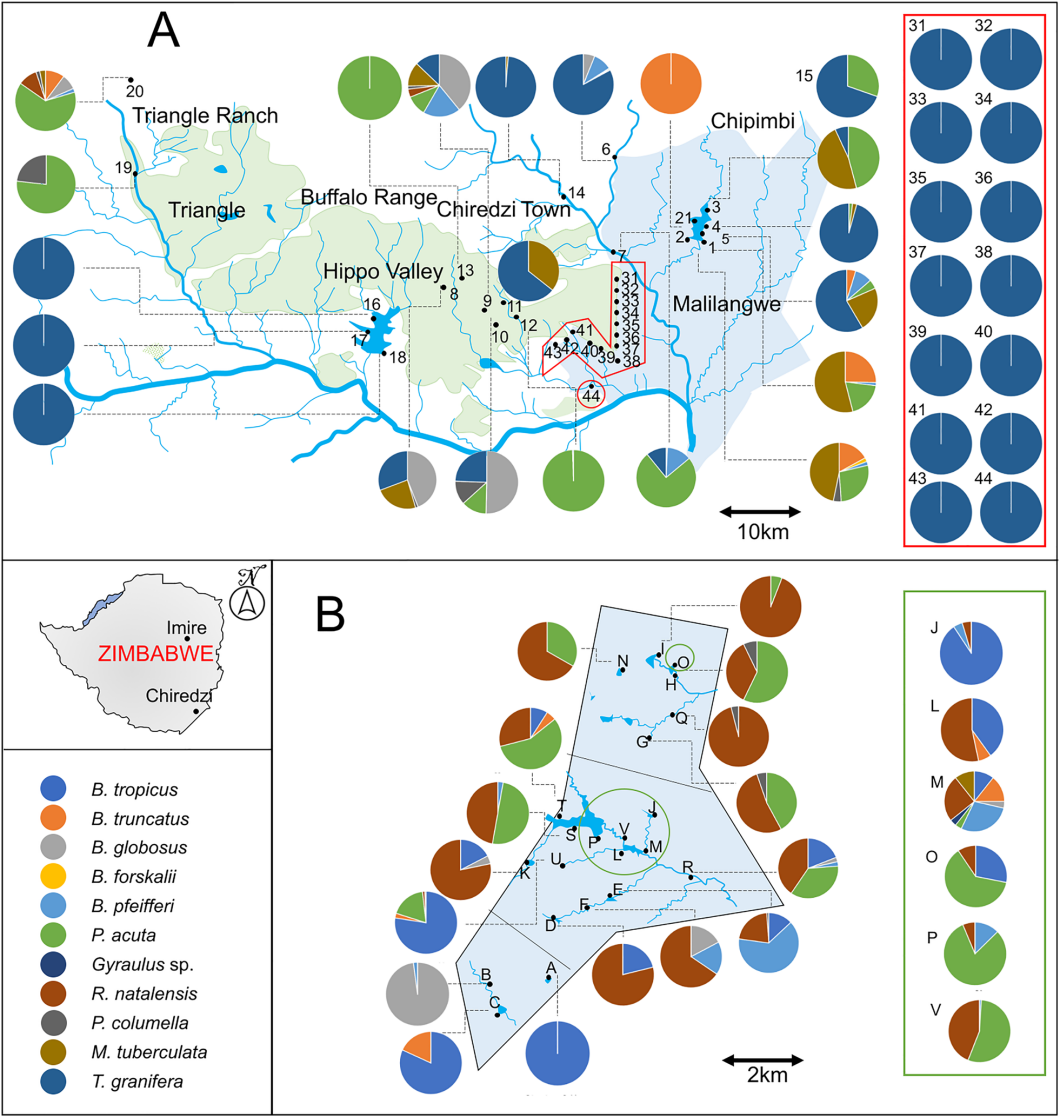
Distribution of snails collected during this study. **A** The sampling map for Chiredzi district including wetlands in the Malilangwe and juxtaposed Hippo Valley and Triangle sugarcane plantations. Most sites have a low diversity of only three or fewer species. Of special note is the widespread coverage by the invasive *Tarebia granifera* (represented by a navy blue color) including sites 6, 14, and 16 to 18 as well as 31 to 44 (pie charts shown in red box as sites are too close to each other). **B** Sampling map of Wedza (Imire). *Radix natalensis* (brown color) was quite widespread and found in almost every site except the three most southerly sites mostly dominated by bulinids (*Bulinus globosus*, *B. tropicus*, and *B. truncatus*). Also widely distributed is *Physella acuta* (green color). Sampling sites in green circle are too close together and are projected on the right-hand side for map. See Additional file 1: Table S2 for the detailed number of snails per site. The bottom left side shows the position of Imire (Wedza) and Chiredzi on the Zimbabwean map

A total of 3209 snails were collected in Chiredzi (*n* = 1883) and Wedza (*n* = 1326). This record, however, excludes *T. granifera*, which was by far the most abundant snail species in Chiredzi with counts often exceeding 2500 individuals per site. *Tarebia granifera* also had the highest prevalence, occurring at 29 out of 35 sampled sites. Moreover, it seems to be most abundant and prevalent in areas with proximity to or run-off from irrigated sugarcane farms. As illustrated in Fig. 3, *T. granifera* is most abundant in sites that drain from Hippo Valley Estates sugarcane fields (e.g., sites 16–18 and 31–44), as well as site 6 (Chipimbi River) and 14 (Chiredzi River). However, sites 9–13 are located in Hippo Valley Estates but are not dominated by *T. granifera*. In Chiredzi we found nearly all 11 freshwater snail species mentioned above, except for *Gyraulus* sp. and *B. tropicus*. Counts for medically and veterinary important snails in Chiredzi are as follows: *B. globosus* (*n* = 305), *B. truncatus* (*n* = 259), *B. forskalii* (*n* = 2), *B. pfeifferi* (*n* = 118), *P. columella* (*n* = 40), and *R. natalensis* (*n* = 23). In addition to *T. granifera*, other invasive species found in Chiredzi included *P. acuta* (*n* = 858) and *M. tuberculata* (*n* = 278). In Wedza, all species mentioned above were observed except for *T. granifera* and *B. forskalii*. Counts for medically and veterinary important snails in Wedza are as follows: *B. globosus* (*n* = 55), *B. truncatus* (*n* = 20), *B. tropicus* (*n* = 466), *B. pfeifferi* (*n* = 86), *P. columella* (*n* = 6), and *R. natalensis* (*n* = 401). For other species we found *P. acuta* (*n* = 288), *Gyraulus* sp. (*n* = 1), and *M. tuberculata* (*n* = 3). See Additional file 1: Table S2 for the detailed number of snail species and snail specimens per site. Here, the data also show that sites with high *T. granifera* abundance harbor relatively low numbers of schistosome-competent snails.

### Identified trematode species released from freshwater snails

**Fig. 4:**
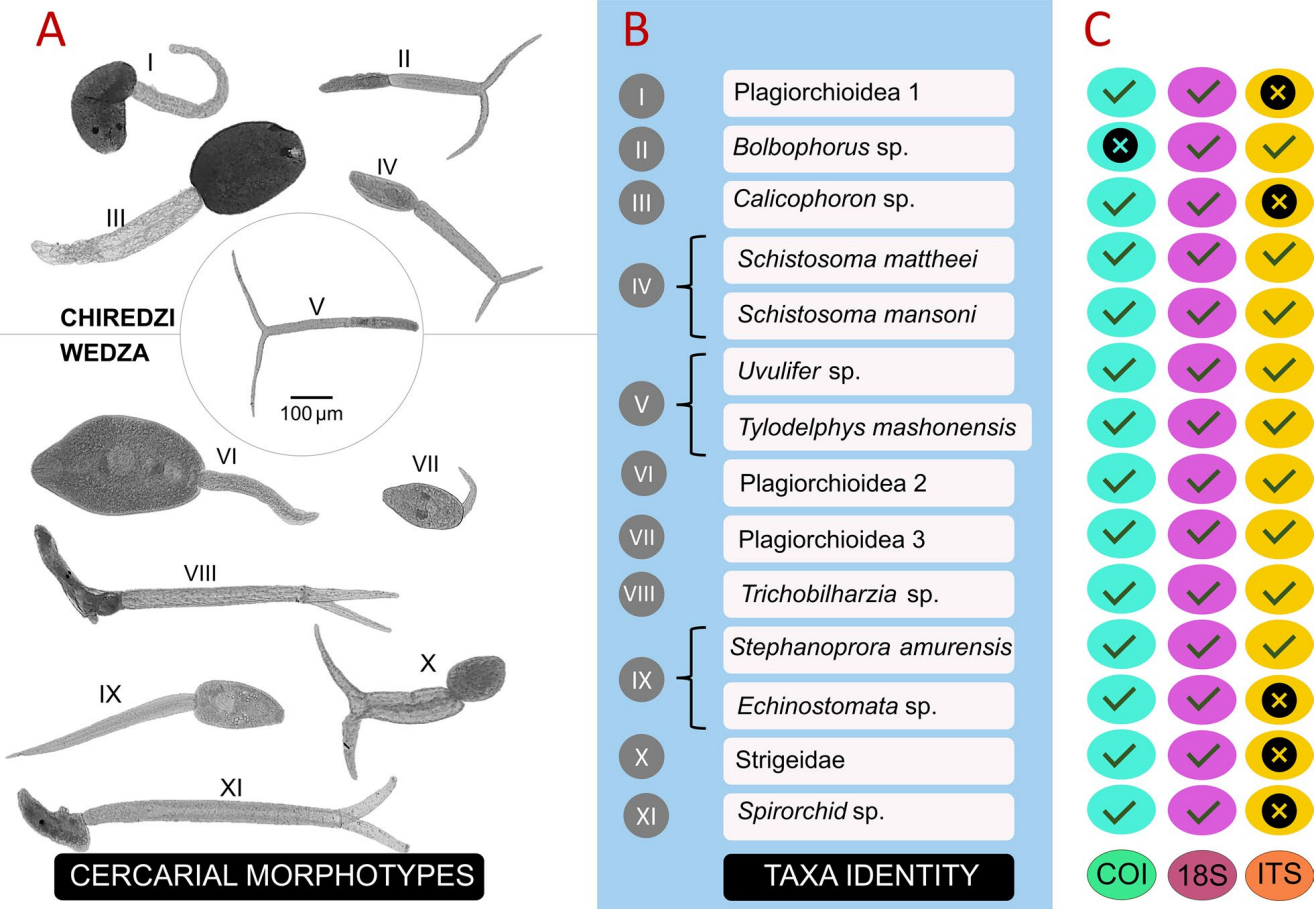
Cercariae identification from snail species in Chiredzi and Wedza. **A** Photographs depict 11 distinct cercarial morphotypes classified based on morphological characteristics following Frandsen and Christensen [37]. Each morphotype is represented by one specimen, with Morph I-IV from Chiredzi, Morph VI-XI from Wedza, and Morph V found in both regions. **B** Molecular genotyping refined the identification, ultimately splitting Morph IV, V, and IX into two, resulting in 14 unique species. **C** Molecular markers (COI, 18S rDNA, and ITS) were employed for cercariae identification, with successful markers denoted by a tick symbol, while unsuccessful ones are marked with an ‘x’

In total, 1743 snail specimens belonging to the following seven species—*B. pfeifferi*, *B. truncatus*, *B. forskalii*, *B. globosus*, *B. tropicus*, *P. columella*, and *R. natalensis*—were tested for shedding. Eleven cercarial morphotypes were recovered. Four were exclusive to Chiredzi: ophthalmocercariae, longifurcate-pharyngeate distome cercariae (subtype I), amphistome type cercariae, and brevifurcate-apharyngeate distome (subtype I) coded as Morph I to Morph IV, respectively (Fig. 4). Longifurcate-pharyngeate distome cercariae (subtype II) (Morph V) was the only overlapping morphotype between Chiredzi and Wedza. The remaining six morphotypes were only exclusive to Wedza: plagiorchiid (subtype I and II), strigea type cercaria, echinostome type cercaria, longifurcate-pharyngeate distome cercaria (subtype III), and brevifurcate-apharyngeate monostome cercaria (subtype III) coded as Morph VI to Morph XI, respectively (Fig. 4).

**Fig. 5:**
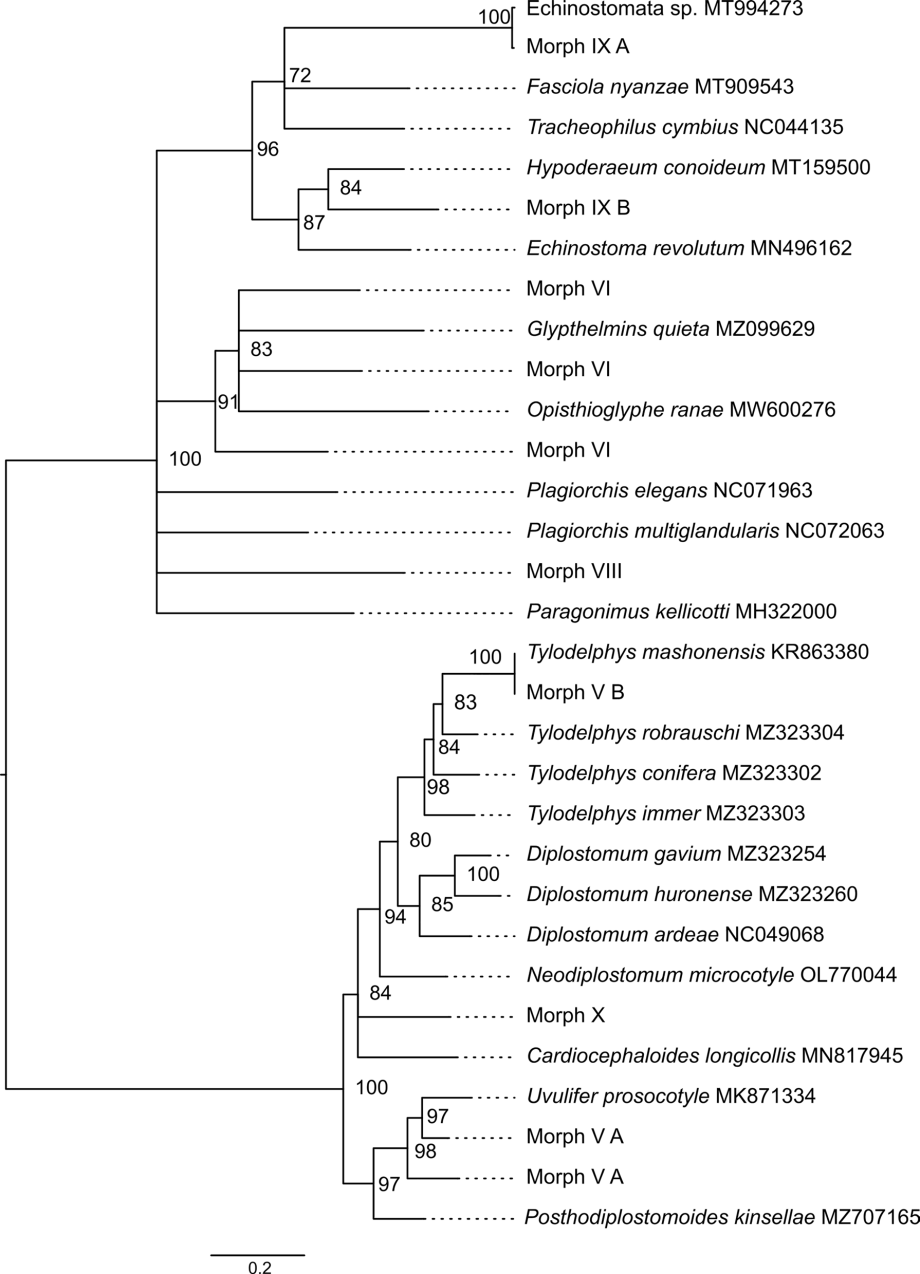
Maximum likelihood phylogenetic tree of superfamilies Diplostomoidea, Echinostomatoidea and Plagiorchioidea using COI (655 bp) and using the General Time Reversible (GTR) model [95] with discrete gamma distribution ([+ G] = 0.59) and invariant sites ([+ I] = 0.34). Nodal support is indicated as bootstrap percentages (10,000 bootstraps). GenBank sequences are displayed with their accession number. Sequences without accession number (labeled as Morphs) were obtained during this study and can be linked to pictures of released larval trematodes (cercariae) shown in Fig. 4. While an attempt was made to identify nine cercarial morphotypes, only two were identified with 100% match due to the barcoding void

For every snail that released cercariae, sequencing was performed on one cercarial specimen per morphotype (Additional file 1: Table S3), and only then were we able to assign sub-morphotypes for ease of interpretation of results. Three markers (COI, 18S, and ITS) were used for the sequence-based analysis where the highest BLAST results and phylogenetics were inferred (Additional file 1: Table S3). At least 14 different trematode species were detected from the 11 cercarial morphotypes. Four morphotypes were identified to species level, i.e. *Schistosoma mattheei* (Morph IV), *S. mansoni* (Morph IV), *Tylodelphys mashonensis* (Morph V), and *Stephanoprora amurensis* (Morph IX). Six morphotypes were identified to genus level, i.e. *Bolbophorus* sp. (Morph II), *Calicophoron* sp. (Morph III), *Uvulifer* sp. (Morph V), *Trichobilharzia* sp. (Morph VIII), *Echinostomata* sp. (Morph IX), and *Spirorchid* sp. (Morph XII). Four species were identified to (super) family level, i.e. Strigeidae (Morph X) and Plagiorchioidea (Morph I, Morph VI, and Morph VII). Figure 4 shows the markers used to determine the taxa identity but see Additional file 1: Table S3 which compiled exact methods used including phylogenetic analysis in Fig. 5 and Additional file 1: Fig. S5).

### Trematode species identified through RD-PCR

In Chiredzi, overall, nine out of 726 snails (1.24%) were shedding. Of these shedding snails, we identified five cercarial morphotypes later differentiated into six different species through molecular genotyping (see above). Next, we used RD-PCR to determine trematode infection in snails. Of the 360 snails from Chiredzi examined by RD-PCR, 44 were found to have a trematode infection, with prevalence in each snail species ranging from 3 to 33%. Four of these infections were *Schistosoma* spp. and two were *Fasciola* sp. infections. The highest trematode infection prevalence was found in *R. natalensis* at 33.3% (11 out of 33) (Table 2). In Wedza, 30 out of 1017 snails (2.95%) were shedding. Of these shedding snails, we identified seven cercarial morphotypes later differentiated into nine different species through molecular genotyping (see above). Of the 616 snails from Chiredzi examined by RD-PCR, 306 were found to have a trematode infection, with prevalence per snail species ranging from 25 to 55%. None of the infections could be attributed to either a *Schistosoma* or *Fasciola* sp. infection. The highest infection rate was detected in *R. natalensis* at 55.4% (159 out of 287), followed by *B. tropicus* at 53.2% (107 out of 201) (Table 2).

**Table 2:** The number of infected snail species based on classical shedding experiments and molecular diagnostic assays

Location	Species	Sampled^a^	Shedding (prevalence)	Tested with PCR	Trematode infection (prevalence)	*Schistosoma* spp. infection (prevalence)	*Fasciola* spp. infection (prevalence)
Chiredzi	*Biomphalaria pfeifferi*	118	6 (5%)	89	11 (12.4%)	2 (18.2%) ^b^	0 (0%)
Wedza	*B. pfeifferi*	86	0 (0%)	57	19 (33.3%)	0 (0%)	0 (0%)
Chiredzi	*Bulinus truncatus*	215	0 (0%)	84	2 (2.8%)	0 (0%)	0 (0%)
Wedza	*B. truncatus*	20	0 (0%)	20	5 (25%)	0 (0%)	0 (0%)
Chiredzi	*B. forskalii*	2	0 (0%)	2	0 (0%)	0 (0%)	0 (0%)
Chiredzi	*B. globosus*	305	3 (1%)	112	15 (13.4%)	2 (13.3%)^c^	0 (0%)
Wedza	*B. globosus*	55	2 (0%)	45	14 (31.1%)	0 (0%)	0 (0%)
Chiredzi	*B. tropicus*	2	0 (0%)	2	0 (0%)	0 (0%)	0 (0%)
Wedza	*B. tropicus*	449	24 (5.4%)	201	107 (53.2%)	0 (0%)	0 (0%)
Chiredzi	*Pseudosuccinea columella*	51	0 (0%)	38	5 (13.2%)	0 (0%)	2 (40%)^d^
Wedza	*P. columella*	6	0 (0%)	6	2 (33.3%)	0 (0%)	0 (0%)
Chiredzi	*Radix natalensis*	33	0 (0%)	33	11 (33.3%)	0 (0%)	0 (0%)
Wedza	*R. natalensis*	401	4 (1%)	287	159 (55.4%)	0 (0%)	0 (0%)

Snail abundance and trematode infections per location (i.e. Chiredzi or Wedza). Biomedically important vector snail species are listed per location along with the number of specimens collected (“Sampled”), the number of snails that shed cercariae in the shedding experiment (“Shedding”), the number of snails for which DNA was extracted for RD-PCRs (“Tested with PCR”), the number of samples that tested positive for a trematode infection (“Trematode infection”), and how many of those trematode infections were either *Schistosoma* spp. (“*Schistosoma* spp. infection”) or *Fasciola* spp. (“*Fasciola* spp. infection”)

^a^All snails sampled were subjected to shedding experiment

^b^*Schistosoma mansoni* recovered from two *B. pfeifferi* snails from Chipimbi River (site 6)

^c^*Schistosoma mattheei* recovered from *B. globosus* snails, one from Chipimbi River (site 6) and other from Masvisvidza Dam (site 20)

^d^*Fasciola nyanzae* recovered from two *P. columella* snails from Gungwa River (site 19)

For those snail specimens with a positive schistosome signal in the RD-PCR, a second, species-specific multiplex PCR test was conducted to discriminate among *Schistosoma* species. *Schistosoma mansoni* was only found at site 6 (Chipimbi River, Chiredzi) with an infection prevalence of 11.1% (2/18) in *B. pfeifferi*. *Schistosoma mattheei* was found in *B. globosus* at site 6 (Chipimbi River) and site 20 (Masvisvidza Dam) with a prevalence of 9.1% (1/11) and 12.5% (1/8), respectively. No *Schistosoma haematobium* infection was found in this study. Trematode infections of two *P. columella* snails (not shedding) from site 19 (Gungwa River, Chiredzi) that appeared positive after RD-PCR (see below) were subjected to COI sequencing. The resultant trematode sequences (399 bp) were 100% identical to *Fasciola nyanzae* from South Africa (“GenBank: ON661099”) and 99.8% to *F. nyanzae* from Zimbabwe (“GenBank: MT909542”).

## Discussion

We studied freshwater snail species and their trematode parasites in a region highly endemic for schistosomiasis. A high snail species diversity including new records, and a plethora of trematode species were found.

### Freshwater snail diversity and spatial distribution

Our study presents a comprehensive malacological survey conducted in two significant biodiversity conservation areas and their adjacent human-influenced zones in Zimbabwe. We identified 11 species of freshwater snails in Chiredzi and Wedza. The snail species diversity in our study aligns with local findings by Schols et al. [30] and Muzarabani et al. [31], reporting 12 and seven species, respectively, in Zimbabwe. Comparable diversity has been reported in other regional studies: eight species in KwaZulu-Natal province, South Africa [44], five species in southwest Ethiopia [45], and eight species in north Cameroon [46]. Furthermore, studies in Denmark recovered 10 species [47], while 15 species were reported from North Rhine-Westphalia, Germany [48].

The identified snail species are *Gyraulus* sp., *Melanoides tuberculata*, *Tarebia granifera*, *Biomphalaria pfeifferi*, *Pseudosuccinea columella*, *Physella acuta*, *Radix natalensis*, *Bulinus globosus*, *Bulinus truncatus*, *Bulinus forskalii*, and *Bulinus tropicus*. Four of these species, *B. pfeifferi*, *B. globosus*, *B. truncatus*, and *B. forskalii*, hold medical significance as intermediate hosts for urinary and intestinal schistosomiasis. The bulinid species, including *B. tropicus*, can also transmit bovine schistosomes [49] and stomach flukes [31]. Other snail species, including *R. natalensis* and *P. columella*, act as intermediate hosts for liver flukes [50]. Four species–*T. granifera*, *M. tuberculata*, *P. columella*, and *P. acuta*–are alien to Zimbabwe (see below).

*Tarebia granifera*, *P. acuta*, and *R. natalensis* emerged as the most widespread species, with *T. granifera* being the most abundant. *Gyraulus* sp. and *B. forskalii* were the rarest and least abundant species. All except one of these species have very recently been documented in previous records for Zimbabwe [28–31], with *T. granifera* being a new record.

*Bulinus forskalii* exclusively inhabited Chiredzi, while *B. tropicus* and *Gyraulus* sp. were unique to Wedza, potentially influencing variations in parasite fauna between the two areas, as discussed in detail below. Found only in Chiredzi, the invasive snail *T. granifera* exhibited particularly high abundance, reaching up to 2500 specimens per site in areas with runoff from sugarcane cultivation. This aligns with existing documentation highlighting the highly eutrophic and fragmented nature of Chiredzi's drainage system [51]–a trait known to attract highly invasive snail species [34, 52]. Our results indeed show that anthropogenically disturbed sites appear more vulnerable to invasive species, in line with other findings [29, 34, 52]. In this case, run-off from sugarcane cultivation appears to facilitate *T. granifera* populations, while native species of medical importance like *Biomphalaria* and *Bulinus* snail species were absent in these sites. Generally, there is higher diversity (at least three species) in the conserved areas, specifically in Lake Malilangwe and in Imire, compared to the cultivated regions mentioned earlier. This observation aligns with literature indicating that human-disturbed ecosystems often witness a decline in macroinvertebrate abundance and diversity [53], although several studies emphasize that schistosome-competent snails thrive in these impacted habitats [54].

### Exotic invasive snails: friend or foe?

Accounts for several exotic invasive snails in southern Africa have been published [29, 55–57]. Aquaculture, recreation, transport, and trade globalization may intentionally or accidentally bolster the transport of exotic invasive freshwater snails, of which some species can become invasive, thereby disrupting local ecosystems [58–60]. One such example is *T. granifera*, a strongly invasive snail species native to regions of Southeast Asia and Oceania [61]. The first report of *T. granifera* in Africa was in 1999 in northern KwaZulu-Natal, South Africa, followed by rapid colonization of the eastern part of the country and neighboring Eswatini [59, 60, 62]. To the best of our knowledge, this is the first report of *T. granifera* identified at species level in Zimbabwe and our sequencing results revealed that our haplotype was (nearly) identical with specimens from Malawi, French Polynesia, Alabama (USA), Thailand, and Timor. Unfortunately, at the present moment, no *T. granifera* COI sequences from South Africa could be found on GenBank making it difficult to trace the source of introduction. In this study, *T. granifera* covered, by the thousands per unit area, nearly all waterbodies in Chiredzi directly linked with the sugarcane plantations. It has been found that *T. granifera* can reach densities of up to 10,000 individuals/m^2^, displacing most endemic snail species, a ‘foe’ in aquatic biodiversity conservation, as was the case in wetlands in Puerto Rico [63], Venezuela [64], and South Africa [55, 65]. However, this potentially creates opportunities for biological control against schistosome/*Fasciola*-competent snail species, thereby becoming a ‘friend’ from a public health perspective [66, 67]. Competitive exclusion might, therefore, explain the absence of endemic snail species at sites where *T. granifera* is present, especially schistosome-competent snails, which were either scarce or entirely absent in the presence of *T. granifera*. Conversely, there was an increased occurrence of schistosome-competent snails in the absence or low numbers of *T. granifera*.

Having a North American origin, the invasive exotic *P. columella* was introduced in South Africa together with exotic aquatic crops in the 1960s [68, 69]. It was described for the first time in Zimbabwe three decades ago [34], and until then only recently reported in the country’s largest reservoir, Lake Kariba [29], as well as in Chiweshe [30]. The *P. columella* isolates from Chiredzi and Wedza were genetically identical to isolates from Lake Kariba (Zimbabwe), South Africa, Australia, Colombia, Egypt, Spain, and the USA, supportive of the ‘flash invasion’ scenario described for this species [69]. *Pseudosuccinea columella* has been reported as a highly compatible host for *F. gigantica* in South Africa [70] and Egypt [50] and for *F. nyanzae* in Zimbabwe [28]. Therefore, *P. columella* is a ‘foe’ associated with the increased transmission of endemic *Fasciola* species–representing a parasite spillback phenomenon whereby a non-indigenous snail host is infected by a native parasite [29]. Also, in this study some *P. columella* specimens were infected with *F. nyanzae*, further emphasizing the increased disease risk posed by this snail (see below).

Finally, *P. acuta*, the third exotic species encountered in this study, was also very widespread, both in Wedza and Chiredzi. Here, we found it in 26 of the 56 sites, with abundance ranging from 1 to 224. It was however never found to be infected. Originating from North America, *P. acuta* has achieved cosmopolitan status through its successful dispersion facilitated by the ornamental aquarium trade [71]. It has proliferated across various global regions, encompassing Africa, Asia, Australia, Europe, and South America [58, 71–74]. Documented instances indicate its displacement of populations of *Bulinus* and *Biomphalaria* species in specific African regions, such as Nigeria, Ghana, and Southern Mozambique [34, 58, 75]. This ecological replacement positions *P. acuta* as a potential ‘friend’ in mitigating schistosomiasis, prompting considerations for its integration into biological control strategies [75, 76]. However, given its invasive tendency, prudent management practices are imperative to prevent *Physella* populations from surpassing ecologically crucial thresholds and therefore becoming a detriment to biological diversity conservation as warned in Lawton et al. [71].

### Biomedically important trematodes with One Health implications

Schistosomiasis and fascioliasis, two diseases of public health and veterinary importance, are of great concern both worldwide and in Zimbabwe. *Biomphalaria pfeifferi* is the primary intermediate host for *S. mansoni* that causes intestinal schistosomiasis in sub-Saharan Africa [77]. Additionally, its relatively high numbers across our sampled regions align with the high prevalence of intestinal schistosomiasis among children, with rates of 43.7% in Chiredzi and 32.3% in Wedza, as reported by Midzi et al. [24]. We found *S. mansoni* in the intermediate snail host *Bi. pfeifferi* in Chiredzi with an overall prevalence of 1.7%. Similarly, we also found *S. mattheei*, a cattle schistosome, infecting *B. globosus* with an overall prevalence of 0.7%. These prevalence levels are on the lower side, but a few infected snail specimens are sufficient for sustaining local transmission. For example, low prevalence of *S. haematobium* in *Bulinus* spp. (1.9%) was recorded in Shamva district, Zimbabwe [78], yet the region has a very high human schistosomiasis prevalence of > 50% [24]. This is contrary to the high overall infection rate of 11.1% of *S. mattheei* in *Bulinus* spp. found in Mazowe district, Zimbabwe [30]. However, the low occurrence could also be attributed to our cross-sectional sampling design, offering only a single snapshot in time. As such, longitudinal studies are needed to account for seasonal dynamics in disease transmission [79]. Although the present study did not find snails infected with *S. haematobium*, the high human prevalence between 10 and 49% in both regions as reported by Midzi et al. [24] and the local presence of *B. globosus* in both districts imply a substantial risk for urogenital schistosomiasis.

Through COI-based molecular barcoding, we found *P. columella* infected with *F. nyanzae* at a prevalence of 2.3%. Our record is, therefore, the third reported district in Zimbabwe after Chiweshe [30] and Kariba [28] with this parasite. *Fasciola nyanzae* is considered to be an exclusive parasite of the common hippopotamus [80, 81]. However, these recent reports partially overlap with livestock areas, raising the potential risk for hybridization with other fasciolids, a phenomenon frequently reported in this group [10, 11]. We also found *Calicophoron* sp. infecting one *B. truncatus* at one site in Chiredzi. This amphistome species of veterinary health concern was reported in Southern Africa, but until now its local intermediate snail host was not yet determined [21].

While no hybrids were discovered in our study, the medically significant trematodes we examined have been previously recognized for their zoonotic potential. These parasites possess the capability to utilize both human and animal reservoir systems, posing significant concerns for their study and control. For instance, certain species of wild rodents serve as reservoirs for *S. mansoni* in endemic areas in Africa and the Caribbean [82, 83]. Additionally, we encountered *S. mattheei*, a cattle parasite, reported to naturally infect free-ranging baboons in Zambia [84]. Therefore, it is imperative to maintain a robust One Health approach to comprehensively assess trematode diversity and hybridization in the sampled region, particularly due to the coexistence of humans and animals in infested areas. Indeed, these monitoring efforts can identify areas of human-animal trematode overlap, informing treatment strategies. For instance, according to Laidemitt et al. [85], treating cattle for the stomach fluke *Calicophoron sukari* could affect human intestinal schistosomiasis prevalence, as the stomach fluke larvae impede the development of *S. mansoni* in *Biomphalaria* snails.

### Similar intermediate hosts miles apart but many different parasites?

Ultimately, our study yielded 15 trematode species as refined by molecular genotyping. Among these, six were previously reported in Zimbabwe: *F. nyanzae* [28], *S. mattheei* [30], *S. mansoni* [30], *T. mashonensis* [86], *Calicophoron* sp., and a Strigeidae species [87]. The remaining nine represent new species records for Zimbabwe: *Bolbophorus* sp., *Trichobilharzia* sp., *Spirorchid* sp., *S. amurensis*, *Uvulifer* sp., three Plagiorchioidea spp., and one Echinostomatoidea sp. None of these parasites have been reported in the study area before, except for *S. mansoni*, which persists even 10 years after the initial report [24] and six rounds of mass deworming [88]. Only five of the 15 trematode species were identified to species level, highlighting the barcoding void. This barcoding void can only be resolved when adult trematode specimens are available, allowing distinct morphological features and life history traits to be linked to genetic information, as demonstrated by Schols et al. [28] and Laidemitt et al. [89]. Although we did not have access to such material, future studies can improve on this and already benefit from our newly established link between the trematode and intermediate host snail–information crucial for completing the parasite's life cycle.

While our trematode diversity aligns with local and regional studies, such as Schols et al. [30] reporting 19 species in three Zimbabwean districts and Outa et al. [90] documenting 17 species in Lake Victoria, Kenya, other regions exhibit even higher diversity. For instance, Lake Takvatn in the sub-Arctic region of Norway reported 24 trematode species [91], and a German nature reserve, Bienener Altrhein, documented 40 species [48]. However, sampling design and efforts differ between these studies, hindering meaningful comparisons.

Numerous factors contribute to trematode distribution, among which is the availability of suitable host snails. Our investigation uncovered notable parasite diversity variations between Wedza and Chiredzi, despite nearly identical snail host diversity as discussed above. Of the 15 trematode species, six were exclusive to Chiredzi, eight to Wedza, and only one species was shared between the two districts. The divergence in parasite transmission patterns in locations with comparable host species diversity is intriguing and highlights the complex factors contributing to geographical heterogeneity, including the widely known influence of aquatic environmental variables [92] and final vertebrate diversity [93]. *Bulinus tropicus*, responsible for the highest trematode diversity (five distinct trematode taxa), was exclusively found in Wedza. It is not uncommon for a single snail species to contribute significantly to trematode diversity. For example, the snail host *Lymnaea stagnalis* exhibited the highest prevalence and diversity, representing 47.6% of species richness across 21 freshwater lakes in Denmark [47]. Our findings, however, contrast with those of Babbitt et al. [94] who reported higher overall trematode diversity among *B. globosus*, *B. ugandae*, and *B. forskalii* (a minimum of five trematode taxa per species) compared to *B. truncatus/tropicus* specimens (two trematode taxa) in the Lake Victoria Basin of Kenya.

## Conclusions

Our study reveals a high snail and trematode species diversity in Chiredzi and Wedza, but the identification of the trematode taxa to species level was seriously hampered by the lack of reference sequences in online databases. The distinct trematode communities encountered across geographic regions with similar freshwater snail hosts, highlights the necessity to explore the effects of other factors involved in disease transmission, including ambient environmental variables and the diversity of final hosts. Finally, the identification of important exotic invasive freshwater species, such as *T. granifera* (a new record in Zimbabwe), *P. columella*, and *P. acuta*, raises concerns about potential alterations in local trematode transmission dynamics, warranting further investigation.

### Supplementary Information

**Additional file 1.**

## Data Availability

All data generated or analyzed during this study are included in this published article and the supplementary materials. Data concerning DNA sequences used in this study are available on NCBI GenBank, with accession codes PP468524-PP468533, PP481879-PP481891, PP556550-PP556562, and PP564875-PP564882. Further inquiries or requests can be directed to the corresponding author.
