# Supplementary material for: One Health monitoring reveals invasive freshwater snail species, new records, and undescribed parasite diversity in Zimbabwe
Source: Parasit Vectors. 2024 May 22;17:234. doi: 10.1186/s13071-024-06307-4 (PMC11110352; doi:10.1186/s13071-024-06307-4)
Supplement: Supplementary file 1 — Additional file 1. [file 13071_2024_6307_MOESM1_ESM.docx]

**One Health monitoring reveals invasive freshwater snail species, new records, and undescribed parasite diversity in Zimbabwe**

**Aspire Mudavanhu^1, 2*^, Ruben Schols^3, 4^, Emilie Goossens^2^, Tamuka Nhiwatiwa^5^, Tawanda Manyangadze^6,7^, Luc Brendonck^2,8^ and Tine Huyse^3^**

^1^ Department of Biological Sciences, Bindura University of Science Education, Bindura, Zimbabwe

^2^ Laboratory of Animal Ecology, Global Change and Sustainable Development, KU Leuven, Leuven, Belgium

^3^ Department of Biology, Royal Museum for Central Africa, Tervuren, Belgium

^4^ Laboratory of Aquatic Biology, KU Leuven Kulak, Kortrijk, Belgium

^5^ Department of Fisheries and Ocean Sciences, School of Agriculture and Fisheries, University of Namibia, Henties Bay, Namibia

^6^ Department of Geosciences, School of Geosciences, Disaster and Development, Faculty of Science and Engineering, Bindura University of Science Education, Bindura, Zimbabwe

^7^ Discipline of Public Health Medicine, College of Health Sciences, University of KwaZulu-Natal, Durban, 4000, South Africa

^8^ Water Research Group, Unit for Environmental Sciences and Management, North-West University, South Africa

* Correspondence: [aspire.mudavanhu@kuleuven.be](mailto:aspire.mudavanhu@kuleuven.be) / [mudavanhu.aspire@gmail.com](mailto:mudavanhu.aspire@gmail.com)

AM: [aspire.mudavanhu@kuleuven.be](mailto:aspire.mudavanhu@kuleuven.be) / [mudavanhu.aspire@gmail.com](mailto:mudavanhu.aspire@gmail.com)

RS: [ruben.schols@africamuseum.be](mailto:ruben.schols@africamuseum.be)

EG: [emilie.goossens@wommersom.com](mailto:emilie.goossens@wommersom.com)

TN: [drtnhiwatiwa@gmail.com](mailto:drtnhiwatiwa@gmail.com)

TM: [manyangadze.tawanda@gmail.com](mailto:manyangadze.tawanda@gmail.com)

LB: [luc.brendonck@kuleuven.be](mailto:luc.brendonck@kuleuven.be)

TH: [tine.huyse@africamuseum.be](mailto:tine.huyse@africamuseum.be)

Additional file 1: Table S1. Site characteristics at all sampling points. Site ID i.e. a number or letter assigned to a site is followed by the full site name. Sites 1-21 and 31-44 are found in Chiredzi whereas site A-V are in Wedza. The water body name in which the site is located, accompanied by observed animal activities are noted. GPS locations are given, along with the water type (e.g. reservoir or river). Site characteristics viz water vegetation, substrate type and depth sampled are also provided.

| **Site ID.** | **Site name** | **Waterbody** | **Observed activities** | **GPS location** | | **Water type** | **Site characteristics** | | **Depth sampled** |
| --- | --- | --- | --- | --- | --- | --- | --- | --- | --- |
|  |  |  |  | **Latitude (South)** | **Longitude (East)** |  | **Water vegetation** | **Substrate** |  |
| 1 | the Harbor | Lake Malilangwe | wildlife | -21.05637017 | 31.8771998 | reservoir | floating water plants | silty/mud | 0-30cm |
| 2 | dam wall | Lake Malilangwe | wildlife | -21.05723422 | 31.87534546 | reservoir | floating water plants | mud | 0-30cm |
| 3 | top reservoir | Lake Malilangwe | wildlife | -21.04250304 | 31.88001756 | reservoir | plants outside water | sand + mud | 0-30cm |
| 4 | Collin's place | Lake Malilangwe | wildlife | -21.03715206 | 31.88510963 | reservoir | floating water plants | muddy | 0-30cm |
| 5 | Reeds/Tsanga | Lake Malilangwe | wildlife | -21.02472187 | 31.8825175 | reservoir | reeds | deep mud | 0-200cm |
| 6 | Chipimbi bridge | Chipimbi River | cattle, humans | -20.9778651 | 31.80593281 | river | reeds + runner float plants | rocky + sand | 0-50cm |
| 7 | Chiredzi river | Chiredzi River | wildlife | -21.04062358 | 31.78400287 | river | grass + reeds | rocky | 0-30cm |
| 8 | workshop canal | hippo valley | sugar cane farming | -21.07605812 | 31.64937808 | irrigation canal | waterlilly, bulrush | mud | 0-20 |
| 9 | irrigation canal | hippo valley | sugar cane farming | -21.07463024 | 31.67567633 | irrigation canal | bulrush, algae | mud | 0-10 |
| 10 | Kisi | hippo valley | sugar cane farming, cattle | -21.07931413 | 31.68670377 | marshland | bulrush, red water fern | mud | 0-20 |
| 11 | Corinth valley farm | hippo valley | sugar cane farming | -21.077329 | 31.688163 | reservoir | bulrush, grass, reeds | not visible | 0-40 |
| 12 | small side canal | hippo valley | sugar cane farming, cattle | -21.082418 | 31.690917 | irrigation canal | bulrush | mud | 0-10 |
| 13 | Railway reservoir | hippo valley | sugar cane farming, cattle | -21.057576 | 31.662549 | reservoir | bies | mud | 0-40 |
| 14 | Bridge Chiredzi River | Chiredzi River | cattle, humans | -21.011886 | 31.746946 | river | bies, reeds | mud | 0-30 |
| 15 | Save bridge | Save river | people swimming, cattle | -20.995055 | 32.157998 | river (+ temporary pools) | algae, reeds | mud, sand or rocks | 0-30 |
| 16 | Mteri dam Nyari lodge | Mteri reservoir | wildlife | -21.123723 | 31.582752 | reservoir | bies | coarse sand | 0-30 |
| 17 | Mteri dam boat launch | Mteri reservoir | wildlife | -21.122803 | 31.570523 | reservoir | bies, grass | coarse sand | 0-30 |
| 18 | Mteri dam wall | Mteri reservoir | wildlife | -21.145037 | 31.590449 | reservoir | bies, grass, frizzled pondweed | coarse sand | 0-30 |
| 19 | Gungwa bridge | Gungwa river | Cattle, humans | -20.990221 | 31.381235 | river | waterlily, water hyacinth | mud | 0 |
| 20 | Samba range dam | Samba range reservoir | hippo, cattle, humans | -20.831443 | 31.331674 | reservoir | grass, cloves, waterlily | mud | 0-30 |
| 21 | reed patch | Lake Malilangwe | fish | -21.048445 | 31.8748890 | reservoir | reeds | mud | 0-20 |
| 31 | HVE drain 1 | HVE drain 1 | sugar cane farming | -21.074620 | 31.800007 | uncemented canal | algae + grass | mud | 0-20 |
| 32 | HVE drain 2 | HVE drain 2 | sugar cane farming | -21.082839 | 31.799773 | uncemented canal | algae + grass | mud | 0-20 |
| 33 | HVE drain 3 | HVE drain 3 | sugar cane farming | -21.092360 | 31.796801 | uncemented canal | algae + grass | mud | 0-20 |
| 34 | HVE drain 4 | HVE drain 4 | sugar cane farming | -21.102368 | 31.796594 | uncemented canal | algae + grass | mud | 0-20 |
| 35 | HVE drain 5 | HVE drain 5 | sugar cane farming | -21.110954 | 31.796449 | uncemented canal | algae + grass | mud | 0-20 |
| 36 | HVE drain 6 | HVE drain 6 | sugar cane farming | -21.116707 | 31.796280 | uncemented canal | algae + grass | mud | 0-20 |
| 37 | HVE drain 7 | HVE drain 7 | sugar cane farming | -21.121725 | 31.796083 | uncemented canal | algae + grass | mud | 0-20 |
| 38 | HVE drain 8 | HVE drain 8 | sugar cane farming | -21.138489 | 31.795474 | uncemented canal | algae + grass | mud | 0-20 |
| 39 | HVE drain 9 | HVE drain 9 | sugar cane farming | -21.139904 | 31.786555 | uncemented canal | algae + grass | mud | 0-15 |
| 40 | HVE drain 10 | HVE drain 10 | sugar cane farming | -21.132656 | 31.773743 | uncemented canal | algae + grass | mud | 0-20 |
| 41 | HVE drain 11 | HVE drain 11 | sugar cane farming | -21.122371 | 31.764817 | uncemented canal | algae + grass | mud | 0-20 |
| 42 | HVE drain 12 | HVE drain 12 | sugar cane farming | -21.126877 | 31.755576 | uncemented canal | algae + grass | mud | 0-20 |
| 43 | HVE drain 13 | HVE drain 13 | sugar cane farming | -21.134517 | 31.750115 | uncemented canal | algae + grass | mud | 0-15 |
| 44 | HVE drain 14 | HVE drain 14 | sugar cane farming | -21.170363 | 31.779204 | uncemented canal | algae + grass | mud | 0-20 |
| A | Chiwawe small dam | Chiwawe small dam | wildlife | -18.5083329 | 31.4818498 | reservoir | Waterlily, bladderwort | mud | 0-30 |
| B | Chiwawe Dam upstream | Chiwawe Dam upstream | wildlife | -18.5134104 | 31.4704021 | river | bladderwort | mud | 0-30 |
| C | Chiwawe Dam Lodge | Chiwawe Dam Lodge | wildlife | -18.5168999 | 31.4717121 | reservoir | bladderwort | mud | 0-30 |
| D | Poplar dam | Poplar dam | wildlife | -18.4927997 | 31.484964 | reservoir | bies, waterlily and ludwigia | mud | 0-30 |
| E | Gerry’s dam | Gerry’s dam | wildlife | -18.4883933 | 31.497301 | reservoir | reed + ludwigia | rocks + mud | 0-30 |
| F | Stream Poplar-Gerry | stream after Poplar dam | wildlife | -18.4912319 | 31.4925182 | river | reed + ludwigia | mud | 0-60 |
| G | Mermaid's dam | Mermaid dam close to the gate | wildlife | -18.4489993 | 31.5079062 | reservoir | oxygen weed, ludwigia | soft sediment | 0-50 |
| H | John’s duck pond | John’s duck pond | birds, wildlife | -18.4417103 | 31.5189341 | reservoir | waterlily, grass | mud | 0-30 |
| I | Booster dam | Booster dam | canoeing | -18.4355231 | 31.5108788 | reservoir | waterlily, reeds, grass | mud | 0-50 |
| J | Number 6 Dam | Number 6 Dam | wildlife | -18.4684386 | 31.5068285 | reservoir | ludwigia, bies, water lily | mud | 0-30 |
| K | Simon’s dam | Simon’s dam | wildlife | -18.4789687 | 31.4793841 | reservoir | bies, waterlily | mud | 0-30 |
| L | Rhino dam | Rhino dam | ducks | -18.4775193 | 31.4991895 | reservoir | bies, waterlily | mud | 0-30 |
| M | Chinyika dam | Chinyika dam | wildlife | -18.478919 | 31.5046711 | reservoir | bies, oxygen weed, water lily, ludwigia | mud | 0-30 |
| N | Mc Johns dam | Mc Johns dam | ducks | -18.4371925 | 31.5025337 | reservoir | oxygen weed fine, oxygen weed regular, waterlily, reed, grass mat | mud | 0-30 |
| O | Kurima dam | Kurima dam | none | -18.4356236 | 31.5156371 | reservoir | grass mat, reed, waterlily, ludwigia | mud | 0-30 |
| P | Sable drinking spot | Numwa dam | sables, wildlife | -18.4727937 | 31.4935189 | reservoir | waterlily, oxygen weed, grass | mud | 0-30 |
| Q | Mermaid river | Mermaid river | wildlife | -18.4370159 | 31.5100044 | river | watercress | mud | 0-30 |
| R | Chinyika river | Chinyika river | wildlife | -18.4830718 | 31.5121816 | river | bies, oxygen weed, water lily, ludwigia | mud | 0-30 |
| S | Numwa dam canoe dock | Numwa dam | wildlife | -18.4720512 | 31.4899044 | reservoir | oxygen weed, reed, grass, water lily | mud | 0-30 |
| T | Numwa dam west | Numwa dam | wildlife | -18.4704655 | 31.4882975 | reservoir | reed, oxygen weed, bies, water lily | mud | 0-30 |
| U | Sam’s dam | Sam’s dam | wildlife | -18.4802455 | 31.4870337 | reservoir | papyrus, bies, water lily, ludwigia | mud | 0-30 |
| V | Numwa river | Numwa river | wildlife | -18.4746408 | 31.500157 | river | reed, bies, ludwigia kind, oxygen weed, water lily, papyrus | mud | 0-30 |

Additional file 1: Table S2. Snail abundance record by site and species. Sites 1-21 and 31-44 are located in Chiredzi whereas sites A-V are in Wedza.

| **Site ID** | **Site name** | ***Bu. tropicus*** | ***Bu. truncatus*** | ***Bu. globosus*** | ***Bu. forskalii*** | ***Bi. pfeifferi*** | ***Ph. acuta*** | ***Gyraulus* sp.** | ***R. natalensis*** | ***Ps. columella*** | ***M. tuberculata*** | ***T. granifera*** |
| --- | --- | --- | --- | --- | --- | --- | --- | --- | --- | --- | --- | --- |
| 1 | Malilangwe dam Harbor | 0 | 16 | 0 | 0 | 1 | 12 | 0 | 0 | 0 | 34 | 0 |
| 2 | Malilangwe Dam wall | 0 | 8 | 0 | 1 | 1 | 13 | 0 | 0 | 2 | 22 | 0 |
| 3 | Malilangwe Dam reservoir | 0 | 0 | 0 | 0 | 0 | 33 | 0 | 0 | 0 | 34 | 5 |
| 4 | Malilangwe Dam Collin's | 0 | 0 | 0 | 1 | 0 | 13 | 0 | 0 | 0 | 16 | 658 |
| 5 | Malilangwe Dam Tsanga | 0 | 10 | 0 | 0 | 19 | 10 | 0 | 0 | 0 | 49 | 124 |
| 6 | Chipimbi River bridge | 0 | 0 | 11 | 0 | 18 | 1 | 0 | 0 | 1 | 0 | 150 |
| 7 | Chiredzi River bridge | 0 | 0 | 1 | 0 | 17 | 97 | 0 | 0 | 0 | 0 | 14 |
| 8 | Workshop canal HVE | 0 | 0 | 93 | 0 | 0 | 0 | 0 | 0 | 3 | 50 | 65 |
| 9 | Irrigation canal HVE | 0 | 0 | 122 | 0 | 60 | 33 | 0 | 13 | 5 | 39 | 40 |
| 10 | Kisi HVE | 0 | 0 | 70 | 0 | 0 | 18 | 0 | 0 | 17 | 0 | 34 |
| 11 | Corinth valley farm HVE | 0 | 0 | 0 | 0 | 0 | 0 | 0 | 0 | 0 | 15 | 27 |
| 12 | Small side canal HVE | 0 | 0 | 0 | 0 | 0 | 172 | 0 | 0 | 0 | 0 | 1 |
| 13 | Railway reservoir HVE | 0 | 0 | 0 | 0 | 0 | 136 | 0 | 0 | 0 | 0 | 0 |
| 14 | Bridge Chiredzi River | 0 | 0 | 0 | 0 | 0 | 0 | 0 | 0 | 0 | 16 | 1200 |
| 15 | Save River bridge | 0 | 0 | 0 | 0 | 0 | 224 | 0 | 0 | 0 | 0 | 514 |
| 16 | Mteri Dam Nyari lodge | 0 | 0 | 0 | 0 | 0 | 0 | 0 | 0 | 0 | 0 | 1616 |
| 17 | Mteri Dam boat launch | 0 | 0 | 0 | 0 | 0 | 0 | 0 | 0 | 0 | 0 | 2898 |
| 18 | Mteri Dam wall | 0 | 0 | 0 | 0 | 0 | 0 | 0 | 0 | 0 | 0 | 2644 |
| 19 | Gungwa River | 0 | 0 | 0 | 0 | 0 | 33 | 0 | 0 | 10 | 0 | 0 |
| 20 | Masvisvidzi River Samba Range | 0 | 10 | 8 | 0 | 2 | 63 | 0 | 10 | 2 | 3 | 0 |
| 21 | Malilangwe Dam reed | 0 | 215 | 0 | 0 | 0 | 0 | 0 | 0 | 0 | 0 | 0 |
| 31 | HVE drain 1 | 0 | 0 | 0 | 0 | 0 | 0 | 0 | 0 | 0 | 0 | 2879 |
| 32 | HVE drain 2 | 0 | 0 | 0 | 0 | 0 | 0 | 0 | 0 | 0 | 0 | 2912 |
| 33 | HVE drain 3 | 0 | 0 | 0 | 0 | 0 | 0 | 0 | 0 | 0 | 0 | 2560 |
| 34 | HVE drain 4 | 0 | 0 | 0 | 0 | 0 | 0 | 0 | 0 | 0 | 0 | 2603 |
| 35 | HVE drain 5 | 0 | 0 | 0 | 0 | 0 | 0 | 0 | 0 | 0 | 0 | 2566 |
| 36 | HVE drain 6 | 0 | 0 | 0 | 0 | 0 | 0 | 0 | 0 | 0 | 0 | 2649 |
| 37 | HVE drain 7 | 0 | 0 | 0 | 0 | 0 | 0 | 0 | 0 | 0 | 0 | 2720 |
| 38 | HVE drain 8 | 0 | 0 | 0 | 0 | 0 | 0 | 0 | 0 | 0 | 0 | 2563 |
| 39 | HVE drain 9 | 0 | 0 | 0 | 0 | 0 | 0 | 0 | 0 | 0 | 0 | 2784 |
| 40 | HVE drain 10 | 0 | 0 | 0 | 0 | 0 | 0 | 0 | 0 | 0 | 0 | 2690 |
| 41 | HVE drain 11 | 0 | 0 | 0 | 0 | 0 | 0 | 0 | 0 | 0 | 0 | 2589 |
| 42 | HVE drain 12 | 0 | 0 | 0 | 0 | 0 | 0 | 0 | 0 | 0 | 0 | 2613 |
| 43 | HVE drain 13 | 0 | 0 | 0 | 0 | 0 | 0 | 0 | 0 | 0 | 0 | 2870 |
| 44 | HVE drain 14 | 0 | 0 | 0 | 0 | 0 | 0 | 0 | 0 | 0 | 0 | 2593 |
| A | Chiwawe Dam north | 198 | 0 | 0 | 0 | 0 | 0 | 0 | 0 | 0 | 0 | 0 |
| B | Chiwawe Dam camp | 0 | 0 | 47 | 0 | 1 | 0 | 0 | 0 | 0 | 0 | 0 |
| C | Chiwawe Dam Lodge | 18 | 4 | 0 | 0 | 0 | 0 | 0 | 0 | 0 | 0 | 0 |
| D | Poplar Dam | 30 | 0 | 0 | 0 | 0 | 0 | 0 | 112 | 0 | 0 | 0 |
| E | Gerry's Dam | 12 | 0 | 0 | 0 | 59 | 0 | 0 | 20 | 1 | 0 | 0 |
| F | Stream Poplar-Gerry | 0 | 0 | 5 | 0 | 5 | 0 | 0 | 19 | 0 | 0 | 0 |
| G | Mermaid's Dam | 0 | 0 | 0 | 0 | 0 | 8 | 0 | 10 | 1 | 0 | 0 |
| H | John's duck pond | 0 | 0 | 0 | 0 | 0 | 8 | 0 | 5 | 1 | 0 | 0 |
| I | Booster Dam | 0 | 0 | 0 | 0 | 0 | 1 | 0 | 16 | 0 | 0 | 0 |
| J | Number 6 Dam | 39 | 0 | 0 | 0 | 2 | 0 | 0 | 2 | 0 | 0 | 0 |
| K | Simon’s Dam | 4 | 0 | 1 | 0 | 0 | 0 | 0 | 18 | 0 | 0 | 0 |
| L | Rhino Dam | 6 | 1 | 0 | 0 | 0 | 0 | 0 | 8 | 0 | 0 | 0 |
| M | Chinyika Dam | 3 | 4 | 1 | 0 | 8 | 1 | 1 | 7 | 0 | 3 | 0 |
| N | Mc Johns Dam | 0 | 0 | 0 | 0 | 0 | 5 | 0 | 10 | 0 | 0 | 0 |
| O | Kurima Dam | 9 | 0 | 0 | 0 | 0 | 20 | 0 | 3 | 0 | 0 | 0 |
| P | Numwa Dam sables | 0 | 0 | 0 | 0 | 8 | 51 | 0 | 4 | 0 | 0 | 0 |
| Q | Mermaid River | 0 | 0 | 0 | 0 | 0 | 0 | 0 | 48 | 2 | 0 | 0 |
| R | Chinyika river | 8 | 0 | 1 | 0 | 1 | 15 | 0 | 17 | 0 | 0 | 0 |
| S | Numwa Dam dock | 0 | 0 | 0 | 0 | 1 | 18 | 0 | 17 | 0 | 0 | 0 |
| T | Numwa Dam west | 12 | 7 | 0 | 0 | 0 | 76 | 0 | 39 | 0 | 0 | 0 |
| U | Sam’s Dam | 127 | 4 | 0 | 0 | 0 | 30 | 0 | 2 | 1 | 0 | 0 |
| V | Numwa River | 0 | 0 | 0 | 0 | 1 | 55 | 0 | 44 | 0 | 0 | 0 |

Additional file 1: Table S3. Identification of cercarial morphotypes retrieved from all shedding snails collected in Chiredzi and Wedza using the identification key of Frandsen & Christensen (1) molecular sequencing. Species that were shed are described according to morphotype (“Cercariae morphotype”) per “Snail species and location of origin” whilst also noting the BLAST results considering sequence similarity, query coverage (QC), and accession length (AL) from all three markers used (“Highest BLAST results from COI, 18S rDNA and ITS markers”) with GenBank accession numbers given for each BLAST result. Comments on whether the species was identified or not are also given (“Taxonomic remarks based on molecular (and morphology) inference”).

| **Cercaria morphotype** | **Snail species and location of origin** | **Highest BLAST results from COI, 18S and ITS markers** | **Taxonomic remarks based on molecular (and morphology) inference** |
| --- | --- | --- | --- |
| Morph I: Ophthalmocercaria. | *Bi. pfeifferi*: Lake Malilangwe: (site 2 and 5). | COI (645 bp): 81.9% similarity with *Plagiorchis* sp. MW519502 (96% QC; 750 bp AL). Only specimen from site 2 was successfully sequenced with COI marker.  18S rDNA (1711 bp): 97.8% similarity with *Nudacotyle undicola* MF538578 (100% QC; 4693 bp AL). Only specimen from site 5 was successfully sequenced with 18S marker. | COI: percentage similarity too low for reliable inference.  18S rDNA: species very closely related to *Nudacotyle undicola* MF538578 based on phylogenetic clustering (Additional file 1: Fig S1). Morphology: presence of ocular spots (Fig. 4) is consistent with the family Nudacotyle.  Since two specimens were used for this morph with two different molecular markers, this species could not be determined but broadly classified as a member of the Plagiorchioidea superfamily. |
| Morph II: Longifurcate-pharyngeate distome subtype I cercaria. | *Bu. truncatus* (1): Lake Malilangwe: (site 5). | 18S rDNA (1544 bp): 99.9% similarity with *Bolbophorus levantinus* AF490576 (100% QC; 1835 bp AL).  ITS (980 bp): 91.6% similar to *Bolbophorus damnificus* KU707947 (96% QC; 1049 bp AL). | 18S rDNA: BLAST results show that this species is very closely related or even identical to *Bolbophorus levantinus* AF490576 but 18S only gives information up to superfamily/family level (Additional file 1: Fig S2).  ITS: clusters with *Bolbophorus* spp. (Additional file 1: Fig S3).  Species therefore identified at the genus level as *Bolbophorus* sp*.* |
| Morph III: Amphistome-type cercaria. | *Bu. truncatus*: Masvisvidzi dam (site 20) | COI (747 bp): 97.5% match with *Calicophoron* sp. specimens from Zimbabwe described in Schols et al. (2) MT994280 (90% QC; 761 bp AL).  18S rDNA (1754 bp): 99.9%) similar to *Gastrothylax crumenifer* JX518985 (100% QC; 1858 bp AL). | COI: BLAST results are sufficiently high and closely match with *Calicophoron* sp. MT994280.  18S rDNA: BLAST results show close relation to *Gastrothylax* crumenifer, but again, 18S rDNA is not suitable for species identification but rather indicates a Paramphistomidae species.  Therefore, the species is identified as *Calicophoron* sp. which is also consistent with the morphology (see Fig. 4) |
| Morph IV A: Brevifurcate-apharyngeate distome subtype I. | *Bu. globosus* (1): Chipimbi River (site 6)  *Bu. globosus* (1): Masvisvidza dam (site 20) | COI (398 bp): 99.7% match with *Schistosoma mattheei* AY157211 (100% QC; 1125 bp AL).  18S rDNA (1465 bp): 100% identical with *S. mattheei* AY157237 (100% QC; 1813 bp AL).  ITS (479 bp): 98.8% similar to *S. mattheei* OR062308 (100% QC; 861 bp AL).  All differences are due to base pair mismatches. | All markers used confirmed this morphotype through BLAST as *S. mattheei*. Morphology: confirms it as a mammalian schistosome species.  Species identified as *S. mattheei*. |
| Morph IV B: Brevifurcate-apharyngeate distome subtype I. | *Bi. pfeifferi*: Chipimbi River (site 6) | COI (741 bp): 96.4% likeness with *Schistosoma mansoni* MG562512 (99% QC; 1029 bp AL). Differences due to base pair mismatches.  18S rDNA (1051 bp): 99.9% identical with *S. mansoni* XR_001974600 (100% QC; 1973 bp AL).  ITS (902 bp): 99.9% match due to one bp deletion with *S. mansoni* AF531314 (100% QC; 966 bp AL). | All markers used confirmed this morphotype through BLAST as *S. mansoni.* Morphology: confirms it as a mammalian schistosome species. |
| Morph V A: Longifurcate-pharyngeate distome subtype II cercaria. | Chiredzi: *Bi. pfeifferi* (2): Chipimbi River (site 6)  Wedza: *Bu. tropicus* (2): Imire (site D (2)). *Bi. pfeifferi* (1): E (1)). | COI (699 bp): 91.1% similar to *Uvulifer prosocotyle* MK871334 (99% QC; 1013 bp AL).  18S rDNA (1359 bp): 99.5% similar with *Uvulifer denticulatus* MG770032 (100% QC; 1653 bp AL).  ITS (991 bp): 99.6% match with *Uvulifer* sp. MK604882 (100% QC; 1238 bp AL). | COI: clusters with *Uvulifer prosocotyle* MK871334 (Fig. 5) yet not strong enough to be considered the same species but indeed the same genus.  18S rDNA: clusters with *Uvulifer* *denticulatus* MG770032 (Additional file 1: Fig S1) and BLAST results are sufficiently high to assign the species to the *Uvulifer* genus.  Species therefore identified as *Uvulifer* sp. |
| Morph V B: Longifurcate-pharyngeate distome subtype II cercaria. | *Bu. tropicus* (4): Imire (sites A (1), D (1), and J (2). | COI (732 bp): 88.7% match with *Tylodelphys robrauschi* MZ323304 (99% QC; 916 bp AL).  18S rDNA (1397 bp): 99.5% match with *Tylodelphys* sp. MH521252 (100% QC; 8032 bp AL). The difference is due to one bp deletion and two mismatches.  ITS (1012 bp): 99.7% match with *Tylodelphys mashonensis* FJ470402 (100% QC; 1083 bp AL). | COI: despite low blast results, phylogenetic analysis identifies the species as *Tylodelphys mashonensis* (Fig. 5).  18S rDNA: affiliates with *Tylodelphys* spp. which both belong to the family Diplostomidae.  ITS: phylogenetic analysis also confirms the species as *T. mashonensis*.  Species identified as *T. mashonensis* due to agreement of at least two markers. |
| Morph VI: Plagiorchiid type I  Possibly 002A by virtue of clustering. | *Bu. tropicus*: Imire (site A (3), D (1) and T (1)) | COI (758 bp): only 81.4% similarity with *Glypthelmins* sp. MW427960 (99% QC; 14200 bp AL).  18S rDNA (1106 bp): 94.3% match with *Auridistomum chelydrae* AY222159 (99% QC; 1875 bp AL).  ITS (810 bp): 86.5% match with *Paramacroderoides echinus* MH041375 (88% QC; 2638 bp AL). | COI: no close phylogenetic similarities but clusters with members of the superfamily Plagiorchioidea (Fig. 5).  18S rDNA: clusters with members of the superfamily Plagiorchioidea (Additional file 1: Fig S1)  ITS: had poor BLAST results and clustering, grouping with members of the superfamily Plagiorchioidea (Additional file 1: Fig S4). Phylogenetic analysis with all three markers agrees that the morphotype is a Plagiorchioidea sp. but the lack of close similarity may reflect a new record. |
| Morph VII: Plagiorchiid type II with dark spots; | *R. natalensis* (2): Imire (site F (1) and G (1)). | COI (855 bp): 100% identical to Plagiorchioidea sp. MT994277 (79% QC; 703 bp AL).  18S (1014 bp): 99.9% similar to Plagiorchioidea sp*.* MT994250 (89% QC; 1107 bp AL).  ITS (695 bp): 96.1% match with Plagiorchiidae sp. AY245700 (100% QC; 3315 bp AL). | COI: clusters with members of the superfamily Plagiorchioidea.  18S rDNA: identifies this morphotype as an unknown member of the superfamily Plagiorchioidea.  ITS: BLAST identifies the species as Plagiorchiidae sp. but phylogenetics clusters with *Orientocreadium indicum* (Orientocreadiidae family) (Additional file 1: Fig S4) but suffers a huge disparity with the accession length therefore cannot be sufficiently identified as such.  Due to these inconsistences the species could not be determined but belongs to the superfamily Plagiorchioidea. |
| Morph VIII : strigea type cercaria. | *R. natalensis* (4) : Imire (site K). | COI (633 bp): 92.3% match with *Trichobilharzia regenti* MN337559 (100% QC; 1089 bp AL).  18S rDNA (1707 bp): 99.7% similar with *Trichobilharzia regenti* AY157218 (100% QC; 1872 bp AL).  ITS (690 bp): 96.8% match with *Trichobilharzia regenti* EF094533 (100% QC; 1870 bp AL). ITS was tested on different specimens from the same snail species and sampling site. | COI: phylogenetically closest to several *Trichobilharzia* species (Additional file 1: Fig S5).  18S rDNA: BLAST identifies this species as *T. regenti*.  ITS: phylogenetically closest to several *Trichobilharzia* species (Additional file 1: Fig S5).  Species therefore identified as *Trichobilharzia* sp. |
| Morph IX A: Echinostome type cercaria. | *Bu. tropicus*: Imire (site A (9) and J (1) | COI (769 bp): 99.5% similar to *Echinostomata* sp. MT994273 (72% QC; 569 bp AL).  18S rDNA (740 bp): 100% identical to Echinostomatoidea sp. MT994245 (100% QC; 1071 bp AL).  ITS (883 bp): 99.6% identical to *Stephanoprora amurensis* MZ412883 (100% QC; 1194 bp AL). | COI: 100% phylogenetic affinity with an identified Echinostomata species recovered from Zimbabwe (GenBank: “MT994273”) (2).  18S rDNA: belongs to the family Echinostomatidae (Additional file 1: Fig S1).  ITS: a sufficiently high BLAST output and strong clustering (Additional file 1: Fig S4) confirms this species as *S.* *amurensis* (family Echinostomatidae). |
| Morph IX B: Echinostome type cercaria. | *Bu. tropicus*: Imire (site J (1)) | COI (682 bp): 84.6% similar to *Hypoderaeum conoideum* MT159500 (97% QC; 1041 bp AL).  18S rDNA (1419 bp): 99.9% identical to *Echinostoma revolutum* OP627676 (100% QC; 3070 bp AL). | COI: identifies this morphotype as *Echinostomata* sp. (Fig. 5).  18S rDNA: Clusters closely with members *Echinostoma* spp. (Additional file 1: Fig S1). Species identified as *Echinostomata* species due to agreement of both markers but different from Morph IX A. |
| Morph X: Longifurcate-pharyngeate distome subtype III cercaria. | *Bu. tropicus* (2): Imire (site U). | COI (497 bp): 87% similarity with *Cardiocephaloides longicollis* MN817945 (100% QC; 985 bp AL).  18S rDNA (1340 bp): 98.7% match with *Ichthyocotylurus erraticus* AJ287526 (100% QC; 1935 bp AL). | COI: poor BLAST results but clusters with the superfamily Diplostomoidea.  18S rDNA and ITS: also cluster with the superfamily Diplostomoidea.  The genus or species could not be determined but belongs to the superfamily Diplostomoidea. |
| Morph XI: Brevifurcate-apharyngeate monostome cercaria. | *Bu. globosus* (2): Imire (site B). | COI (715 bp): 84.9% similarity with *Spirorchid* sp. AY829242 (84% QC; 723 bp AL).  18S rDNA (1369 bp): 99.3% similar to *Spirorchid* sp. AY829255 (100% QC; 1701 bp AL). | COI: clusters closely with *Spirorchid* sp. (Spirorchiidae). Additional file 1: Fig S6  18S rDNA: clusters with members of the family Spirorchiidae (Additional file 1: Fig S2).  Based on two markers this species is assigned to the *Spirorchid* genus. |


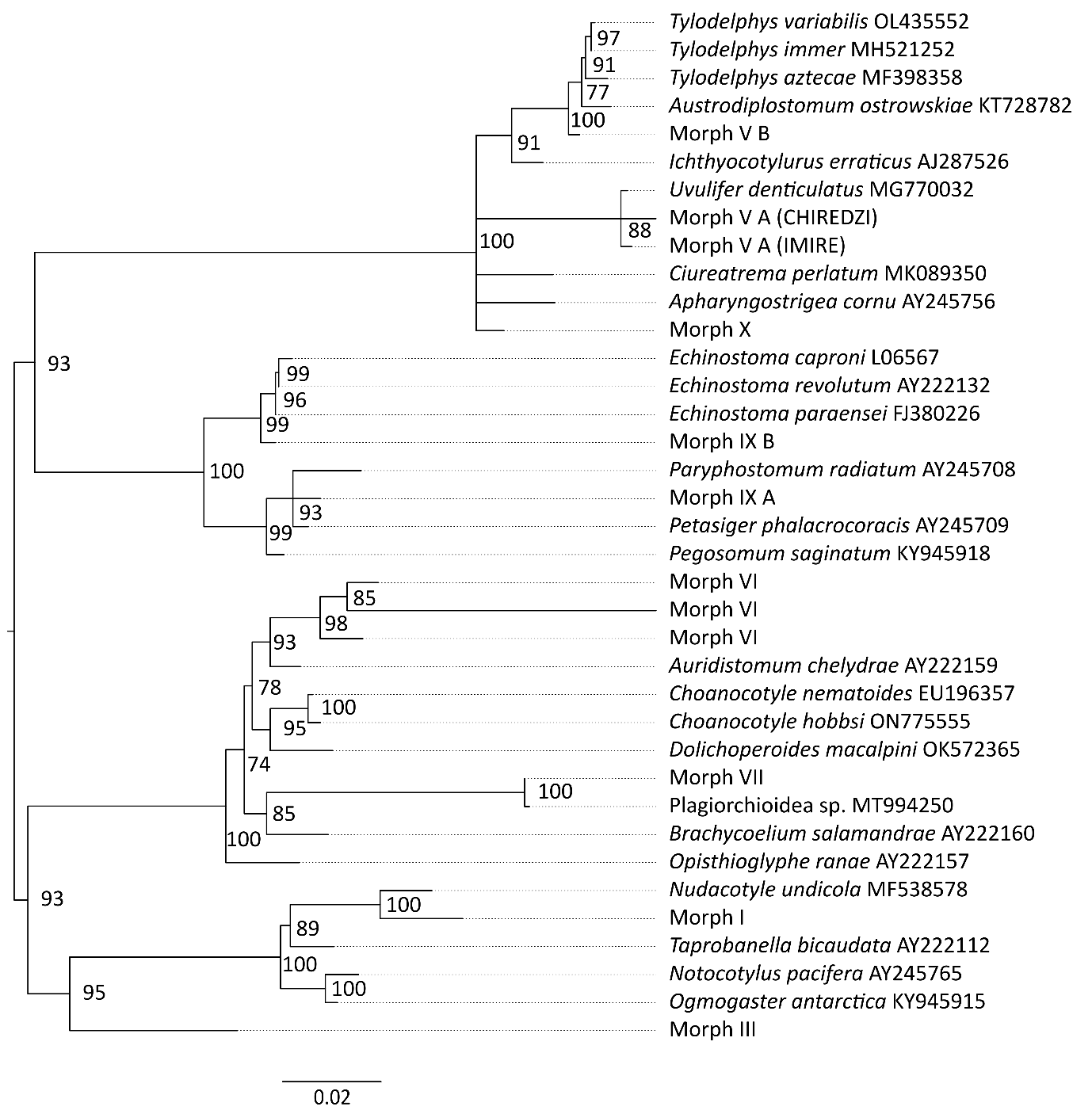


Additional file 1: Fig. S1. Maximum likelihood phylogenetic tree of superfamilies Diplostomoidea, Echinostomatoidea and Plagiorchioidea using 18S rDNA (1563 bp) and using the General Time Reversible (GTR) model (3) with discrete Gamma distribution ([+G] = 0.42) and invariant sites ([+I] = 0.50). Nodal support is indicated as bootstrap percentages (10,000 bootstraps). GenBank sequences are displayed with their accession number (not italicized). Sequences without accession number (labelled as Morphs) were obtained during this study.


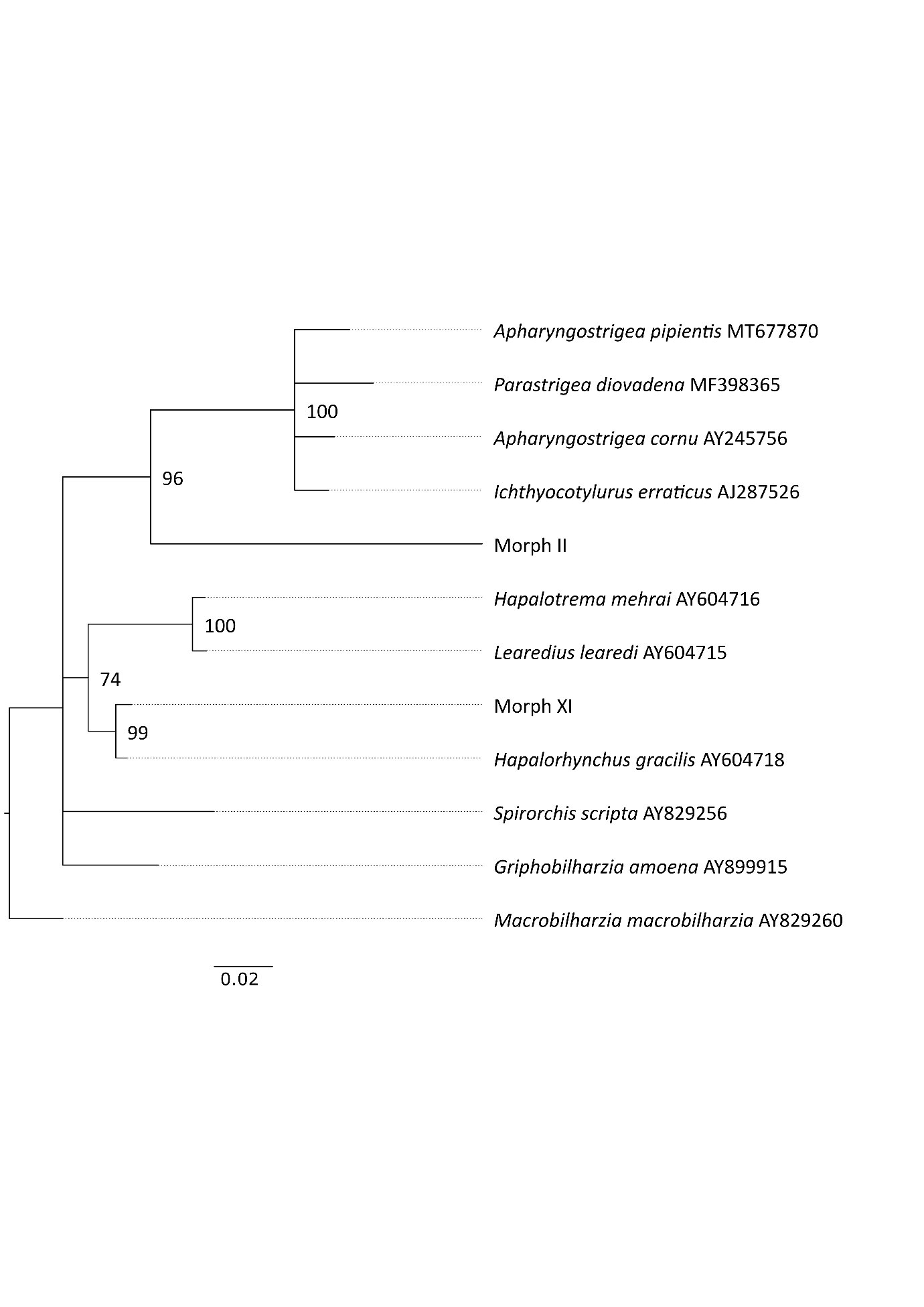


Additional file 1: Fig. S2. Maximum likelihood phylogenetic tree of superfamily Diplostomoidea using 18S rDNA (1702 bp) and using the GTR model with discrete Gamma distribution ([+G] = 0.13). Nodal support is indicated as bootstrap percentages (10,000 bootstraps). GenBank sequences are displayed with their accession number (not italicized). Sequences without accession number, labelled as Morph II and Morph XI, were obtained during this study.


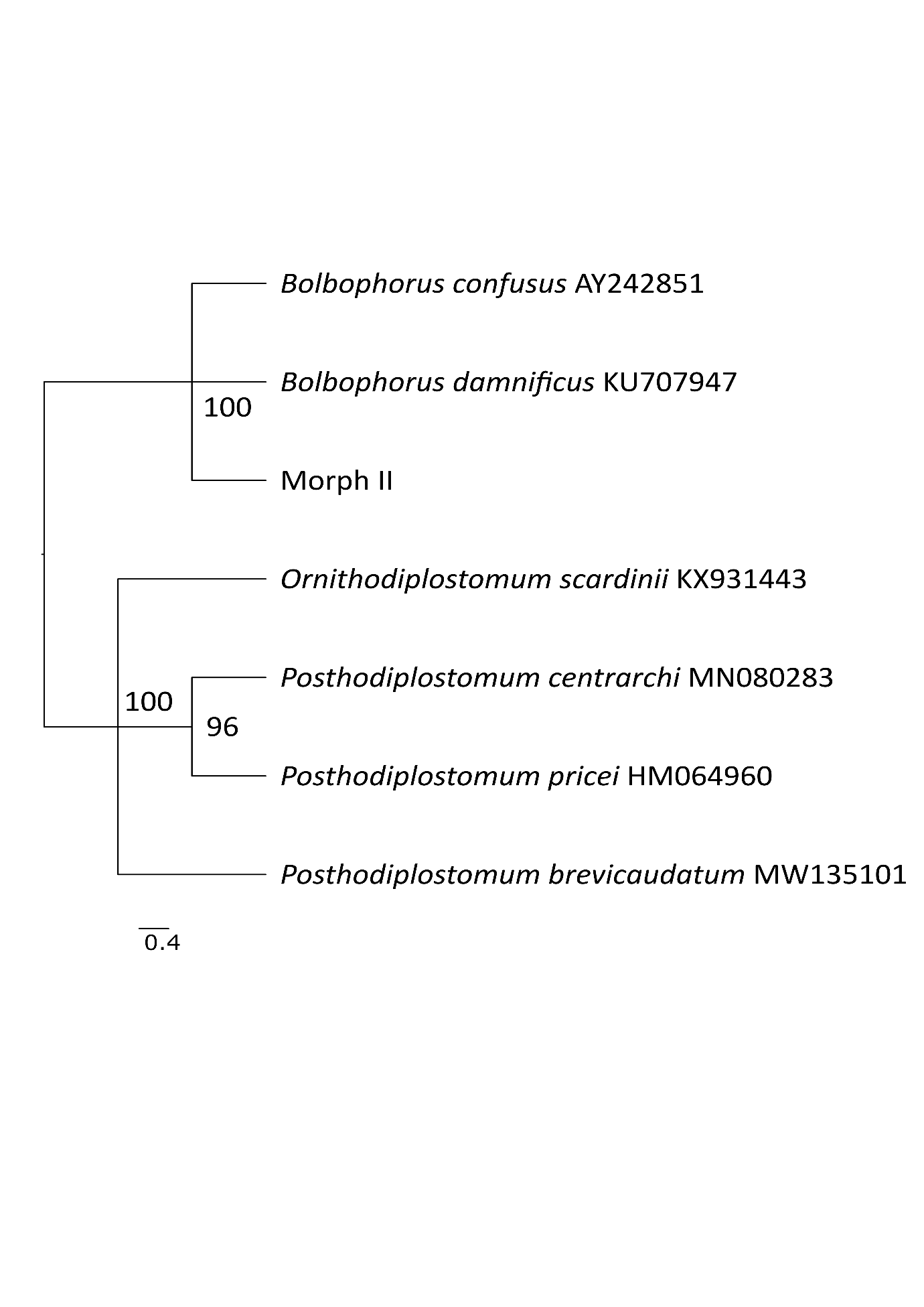


Additional file 1: Fig. S3. Maximum likelihood phylogenetic tree of superfamily Diplostomoidea using ITS (1009 bp) and using the GTR model with discrete Gamma distribution ([+G] = 0.34). Nodal support is indicated as bootstrap percentages (10,000 bootstraps). GenBank sequences are displayed with their accession number (not italicized). The sequence without accession number, labelled as Morph II, was obtained during this study.


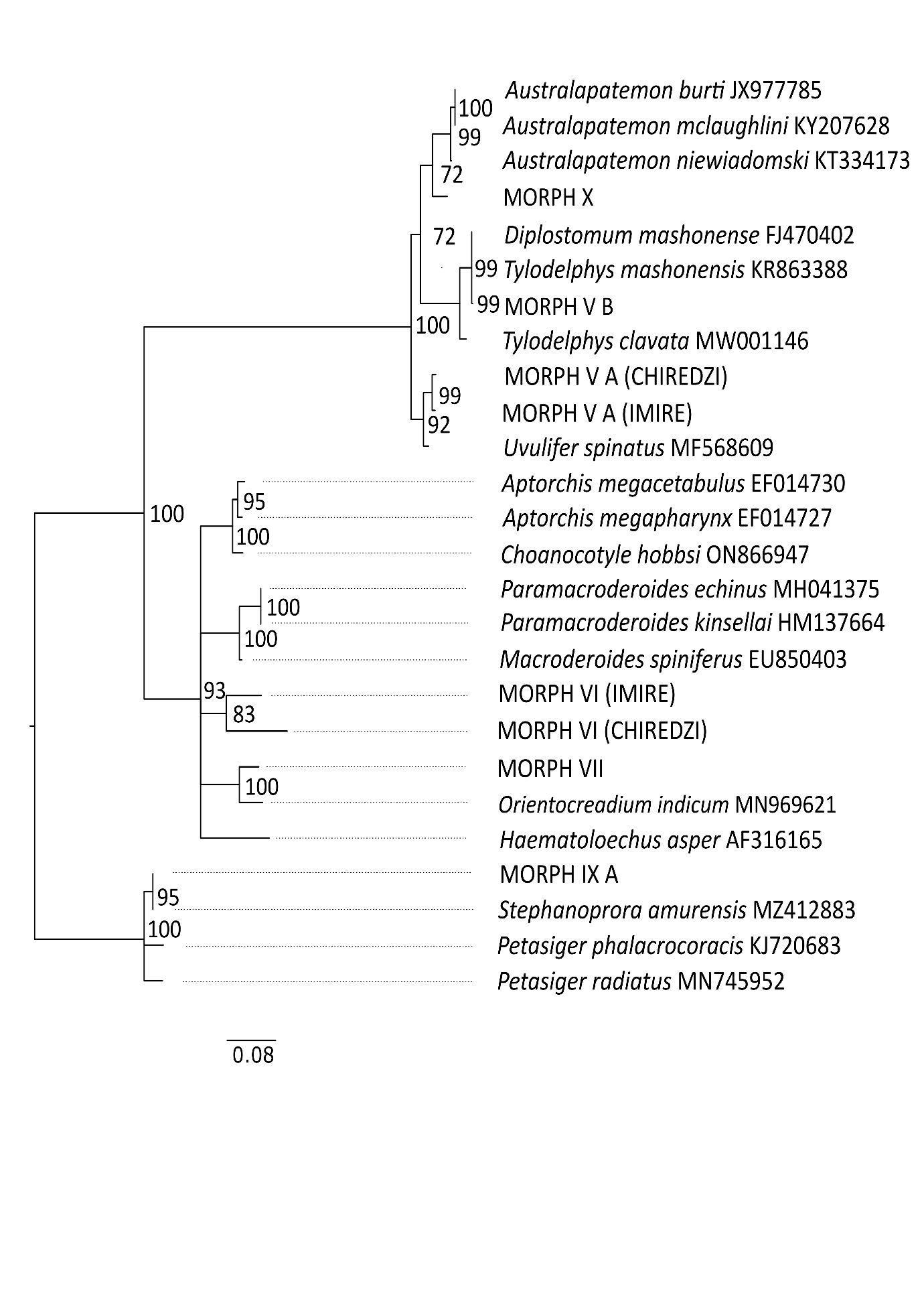


Additional file 1: Fig. S4. Maximum likelihood phylogenetic tree of superfamilies Diplostomoidea, Echinostomatoidea and Plagiorchioidea using ITS (664 bp) and using the GTR model with discrete Gamma distribution ([+G] = 0.47). Nodal support is indicated as bootstrap percentages (10,000 bootstraps). GenBank sequences are displayed with their accession number (not italicized). Sequences without accession number (labelled as Morphs) were obtained during this study.


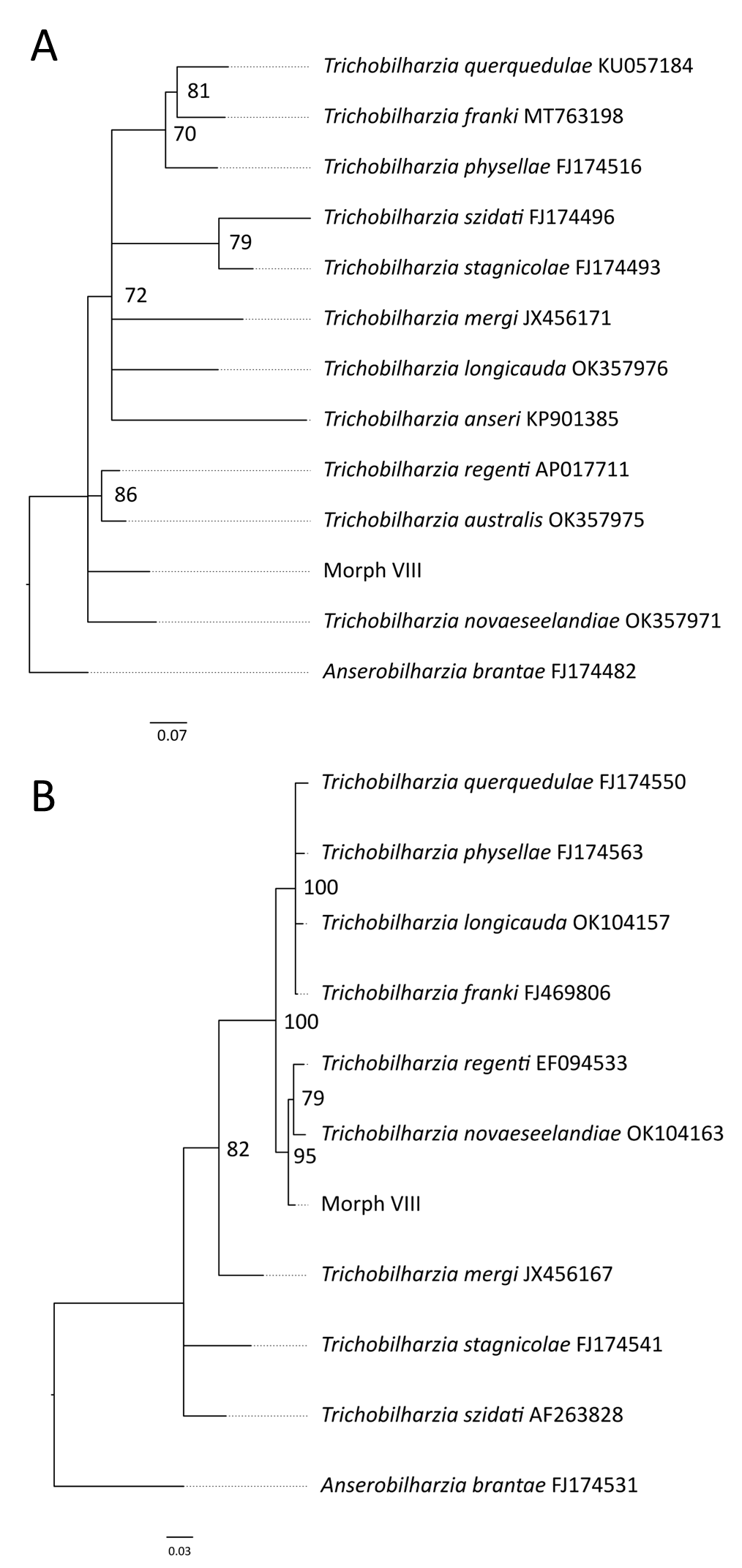


Additional file 1: Fig. S5. Maximum likelihood phylogenetic trees of *Trichobilharzia* spp. using COI (655 bp) sequences (A) and ITS (1206 bp) sequences (B). Hasegawa-Kishino-Yano (HKY) model (4) with discrete Gamma distribution ([+G] = 0.59) and invariant sites ([+I] = 0.34) was selected for the COI dataset while HKY with discrete Gamma distribution ([+G] = 0.21) was selected for the ITS dataset. Bootstrap values (10,000 replicates) that are above 70 are shown next to the branches. GenBank sequences are displayed with their accession number (not italicized). Sequences without accession number (labelled as Morph VIII) were obtained during this study and can be linked to pictures of released larval trematodes (cercariae) shown in Fig. 3.


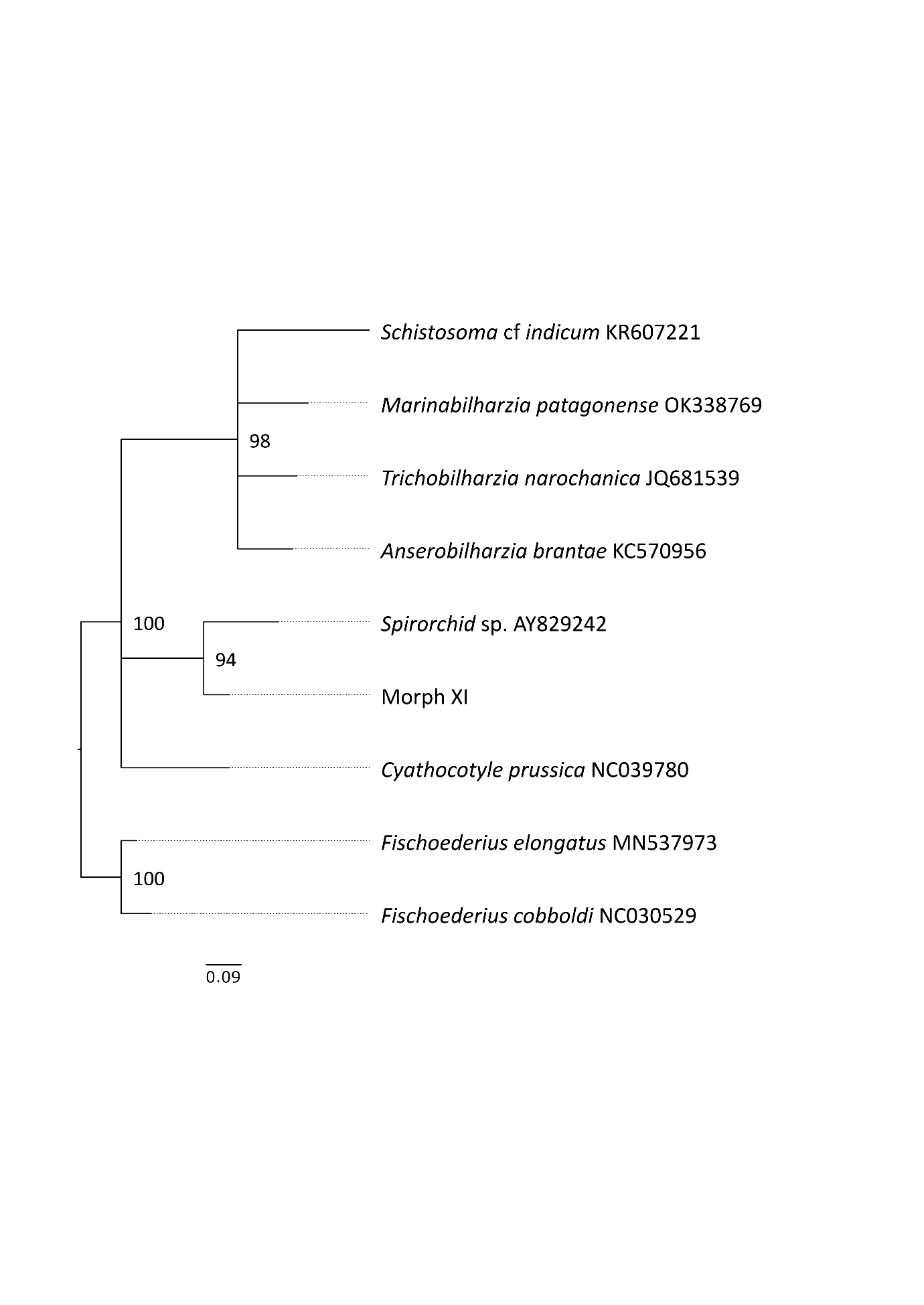


Additional file 1: Fig. S6. Maximum likelihood phylogenetic tree of the family Spirorchiidae using COI (719 bp) and using the GTR model with discrete Gamma distribution ([+G] = 1.53) and invariant sites ([+I] = 0.41). Nodal support is indicated as bootstrap percentages (10,000 bootstraps). GenBank sequences are displayed with their accession number (not italicized). The sequence without accession number, labelled as Morph XI, was obtained during this study.

**References**

1. Frandsen F, Christensen NO. An introductory guide to the identification of cercariae from African freshwater snails with special reference to cercariae of trematode species of medical and veterinary importance. Acta Trop. 1984;41(2):181–202.

2. Schols R, Mudavanhu A, Carolus H, Hammoud C, Muzarabani KC, Barson M, et al. Exposing the barcoding void: An integrative approach to study snail-borne parasites in a One Health context. Front. Vet. Sci. 7:605280.

3. Nei M, Kumar S. Molecular Evolution and Phylogenetics. Oxford, New York: Oxford University Press; 2000. 348 p.

4. Hasegawa M, Kishino H, Yano T. Dating of the human-ape splitting by a molecular clock of mitochondrial DNA. J Mol Evol. 1985;22(2):160–74.
